# Supplementary material for: Design, Synthesis and Investigation of the Potential Anti-Inflammatory Activity of 7-O-Amide Hesperetin Derivatives
Source: Molecules. 2019 Oct 11;24(20):3663. doi: 10.3390/molecules24203663 (PMC6832651; doi:10.3390/molecules24203663)
Supplement: Supplementary file 1 [file molecules-24-03663-s001.pdf]

Supporting Information

Design, synthesis and investigation of potential anti-inflammatory activity of 7-O-amide hesperetin derivatives

Yilong Zhang [1,2,3,†], **Yan Zheng** [1,2,3,†], **Wen Shi** [1,2,3], **Yahui Guo** [1,2], **Tao Xu** [1,2,3], **Zeng Li** [1,2,3], **Cheng Huang** [1,2,3,*], **Jun Li** [1,2,3,*]

1 The Key Laboratory of Major Autoimmune Diseases, Anhui Province, Anhui Institute of Innovative Drugs, School of Pharmacy, Anhui Medical University

2 The key laboratory of Anti-inflammatory of Immune medicines, Ministry of Education

3 Institute for Liver Diseases of Anhui Medical University, Anhui Medical University, Hefei 230032, China

* Correspondence: lj@ahmu.edu.cn, Jun Li, Professor of Pharmacology; huangcheng@ahmu.edu.cn, Cheng Huang, Associate Professor of Pharmacology. School of Pharmacy, Anhui Medical University, 81 Meishan Road, Hefei, Anhui Province, 230032, China. Tel. /fax: +86 551 65161001.

† These authors contributed equally to this paper.

HRMS, ^{1}H NMR and ^{13}C NMR spectra for the compounds.

Figure S0. ^{1}H NMR spectra for heperidin **:**

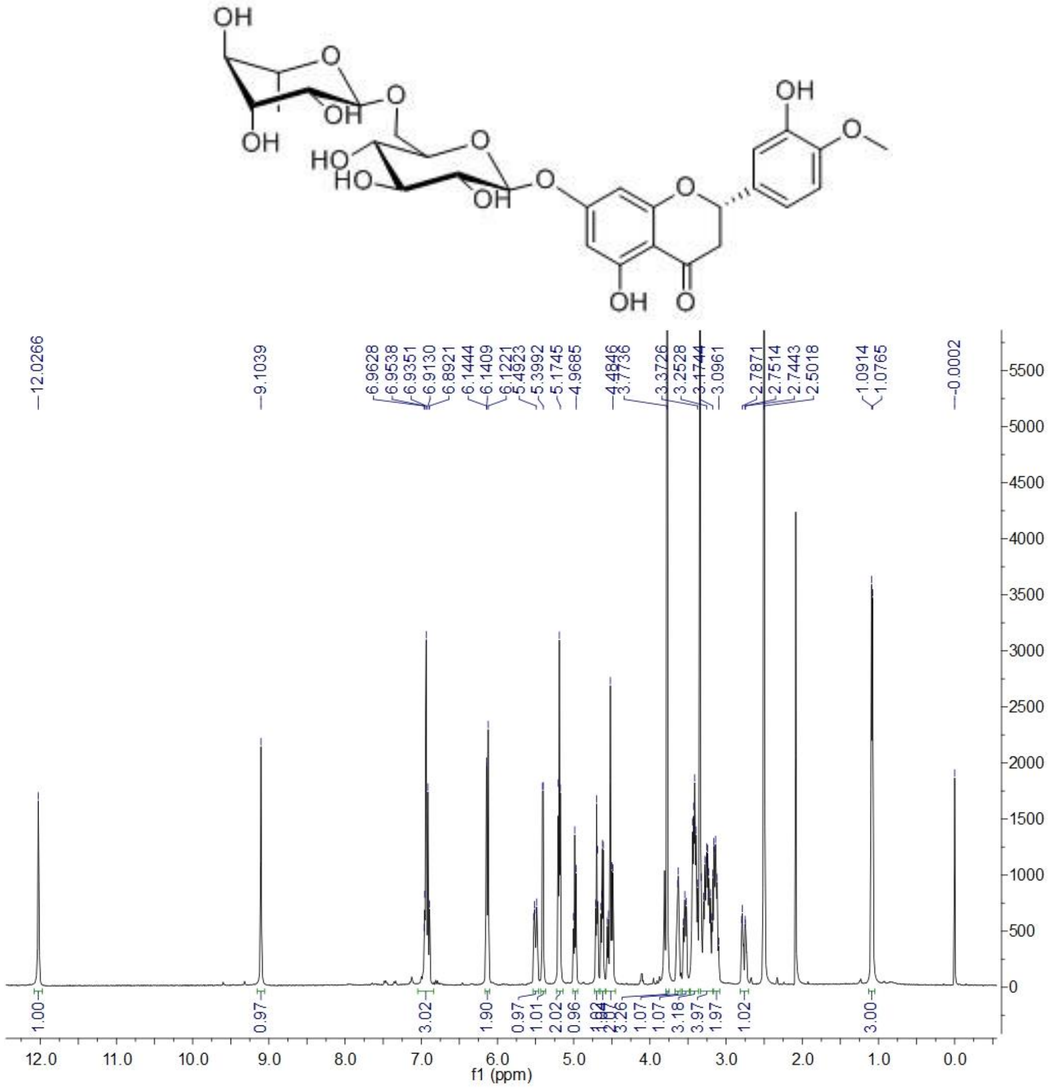

Hesperidin, $C_{28}H_{34}O_{15}$, ^{1}H NMR (400 MHz, DMSO) δ 12.03 (s, 1H, 5-OH), 9.10 (s, 1H, 3′-OH), 7.05 – 6.84 (m, 3H, 2′-H, 5′-H, 6′-H), 6.16 – 6.10 (m, 2H, 6-H, 8-H), 5.50 (dd, *J* = 12.1, 3.1 Hz, 1H, 2-H), 5.41 (d, *J* = 4.9 Hz, 1H), 5.19 (t, *J* = 5.5 Hz, 2H), 4.99 (t, *J* = 6.7 Hz, 1H), 4.70 (t, *J* = 5.0 Hz, 1H), 4.63 (dd, *J* = 7.4, 4.4 Hz, 1H), 4.58 – 4.45 (m, 2H), 3.77 (s, 3H, OCH_3), 3.64-3.62 (m, 1H), 3.57 – 3.48 (m, 1H), 3.47 – 3.36 (m, 3H), 3.31 – 3.19 (m, 4H), 3.19 – 3.07 (m, 2H), 2.77 (dd, *J* = 17.2, 2.9 Hz, 1H, 3-H), 1.08 (d, *J* = 5.9 Hz, 3H, CH_3).

Figure S1. HRMS, ^{1}H NMR and ^{13}C NMR spectra for the compound **1 :**

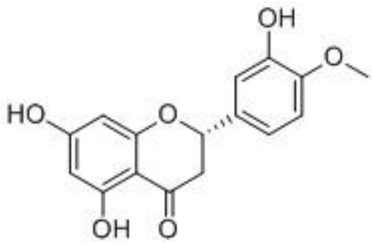

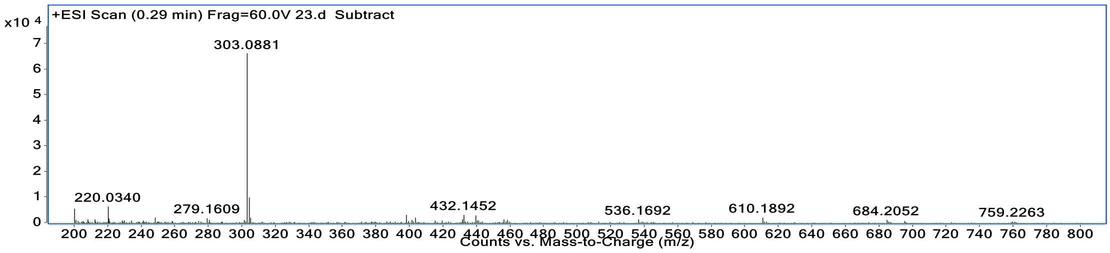

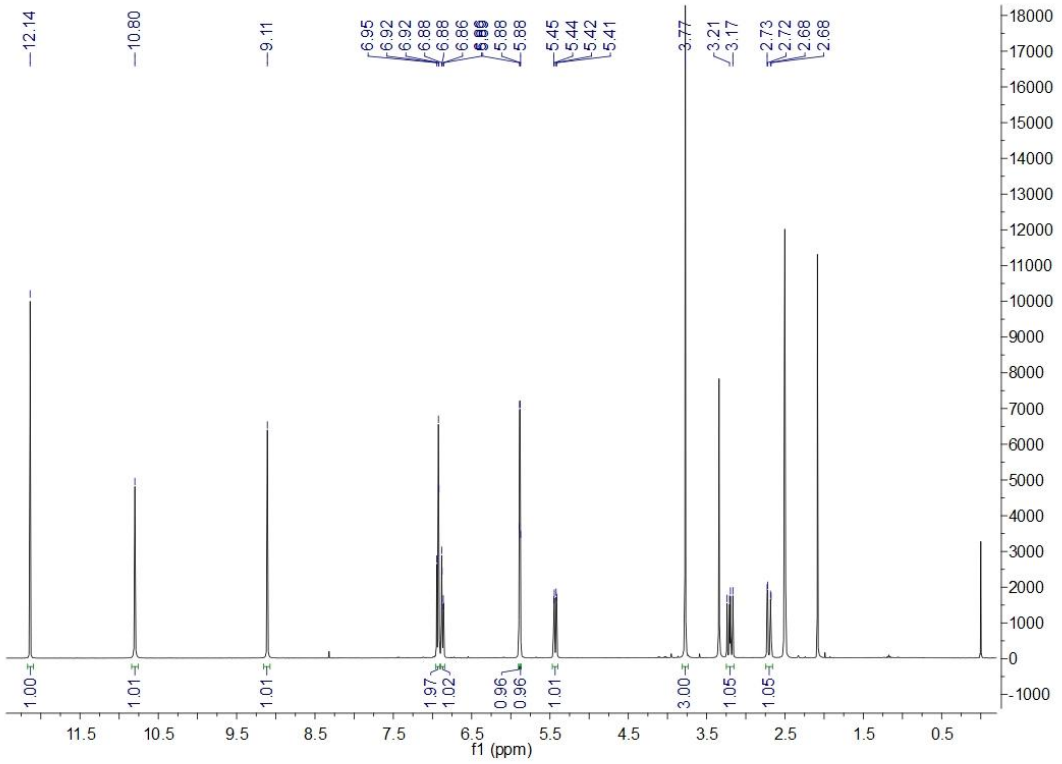

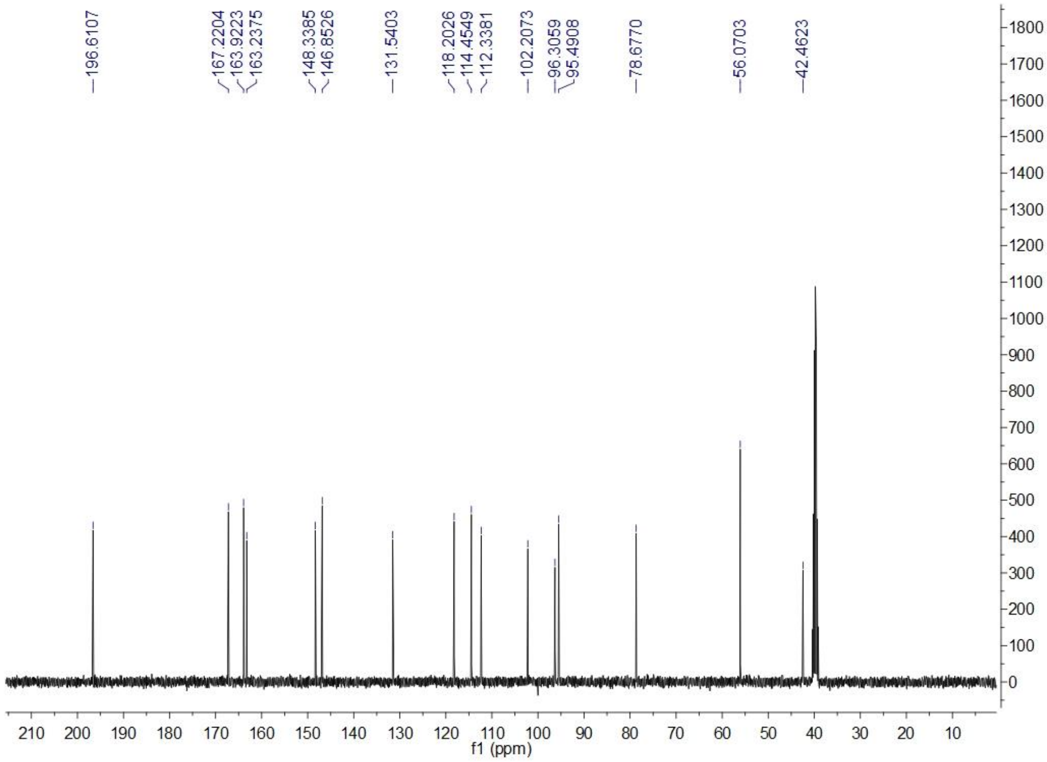

Figure S2. HRMS, ^{1}H NMR and ^{13}C NMR spectra for the target compound **2**

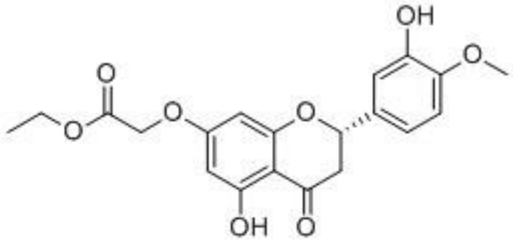

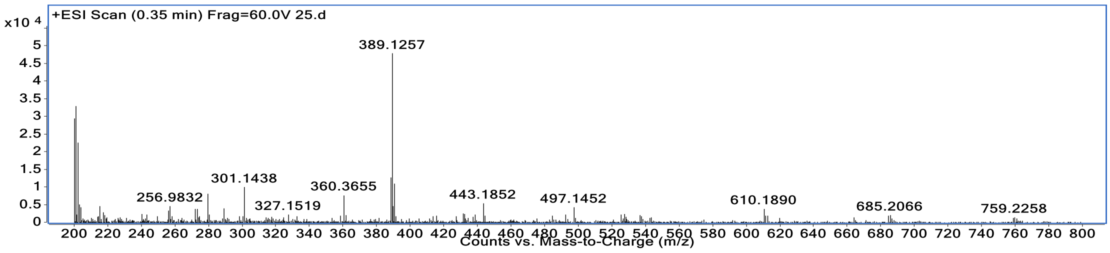

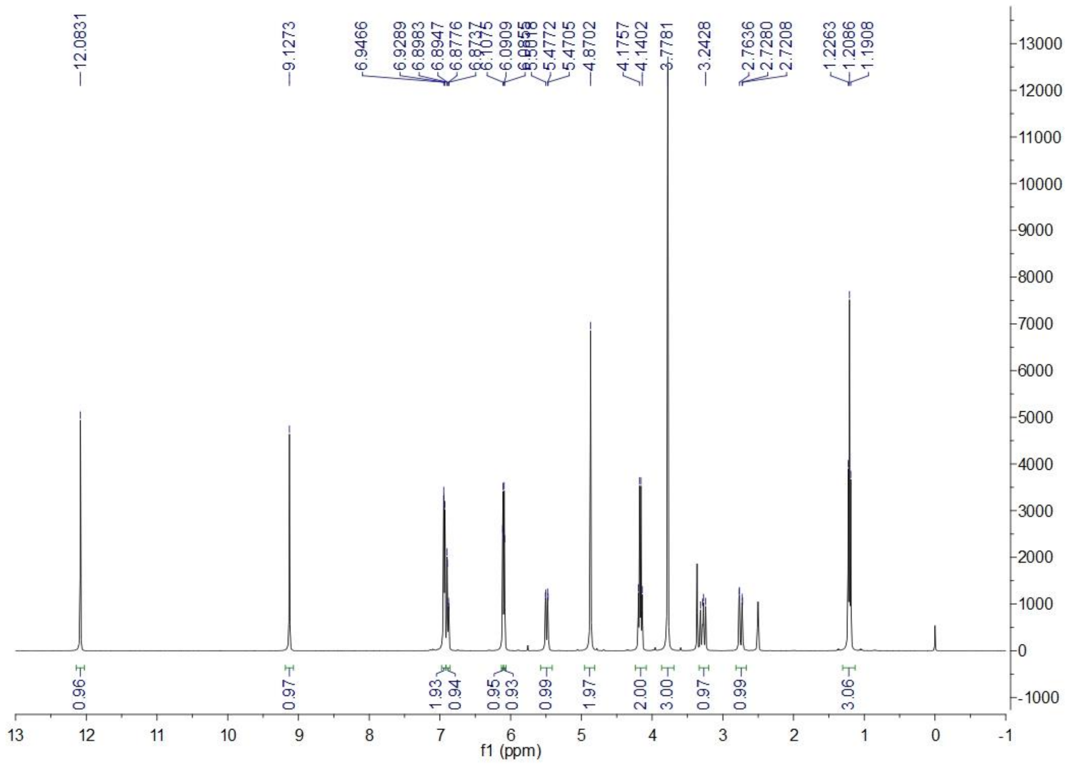
12.0831
9.1273
6.9466
6.9289
6.8983
6.8947
6.8776
6.8737
6.1075
6.0909
5.4772
5.4705
4.8702
4.1757
4.1402
3.7781
3.2428
2.7636
2.7280
2.7208
1.2263
1.2086
1.1908
0.96
0.97
1.93
0.94
0.95
0.93
0.99
1.97
2.00
3.00
0.97
0.99
3.06
f1 (ppm)

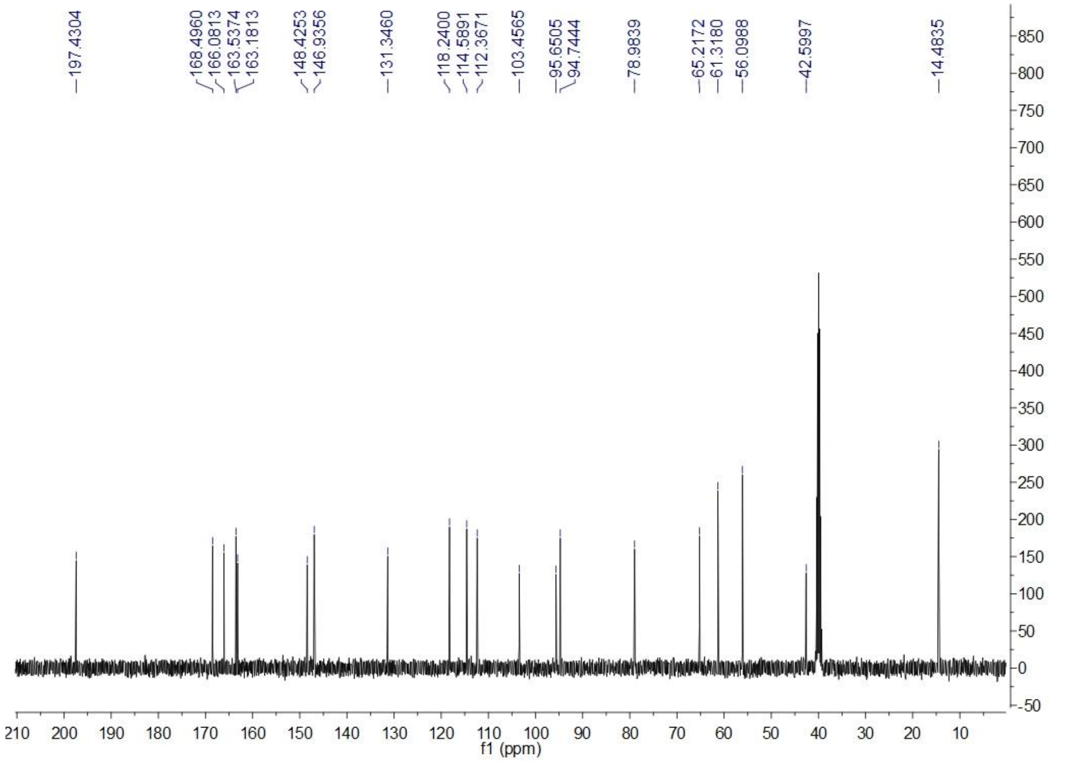
197.4304
168.4960
166.0813
163.5374
163.1813
148.4253
146.9356
131.3460
118.2400
114.5891
112.3671
103.4565
95.6505
94.7444
78.9839
65.2172
61.3180
56.0988
42.5997
14.4835
f1 (ppm)

Figure S3. HRMS, ^{1}H NMR and ^{13}C NMR spectra for the target compound **3:**

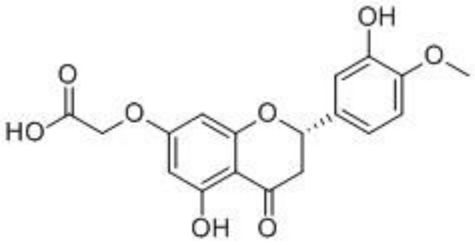

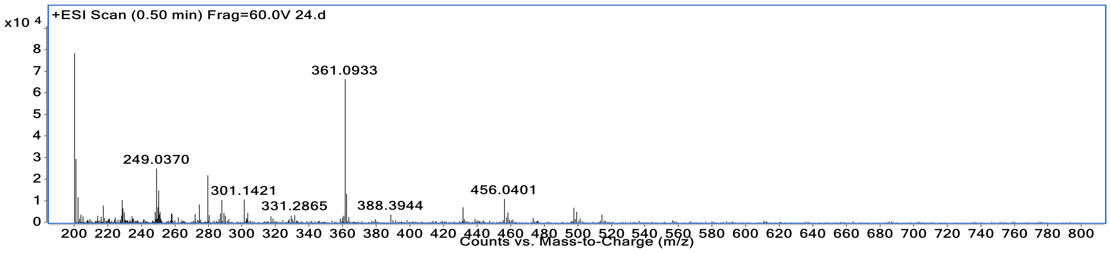

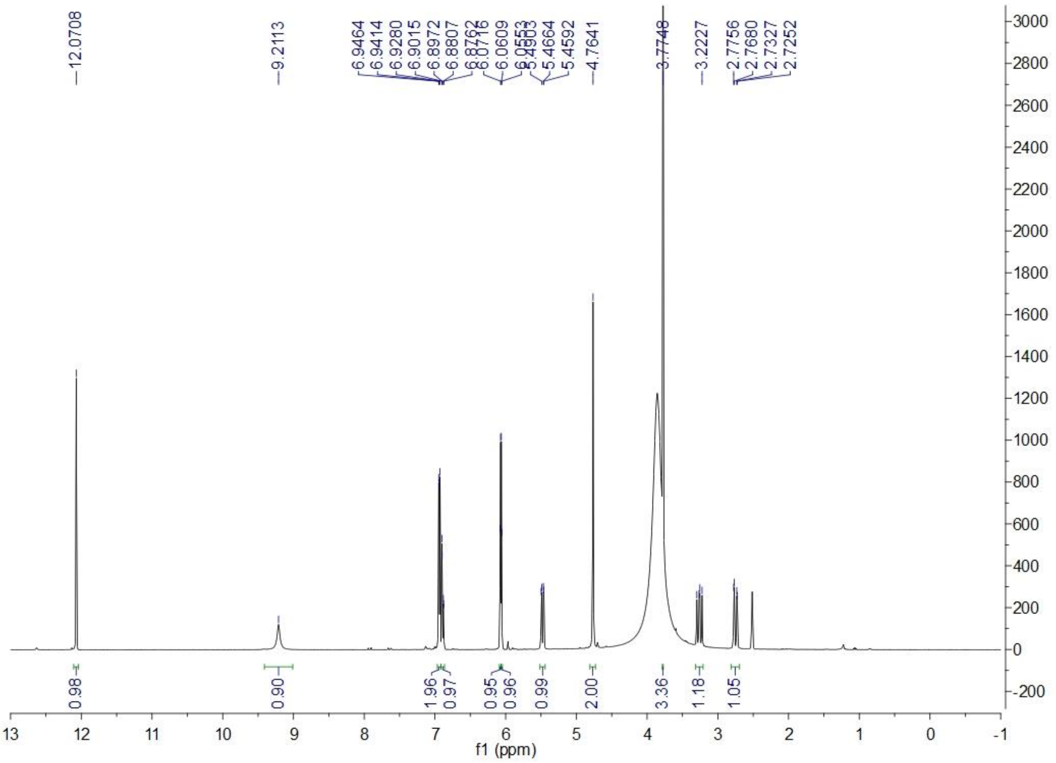

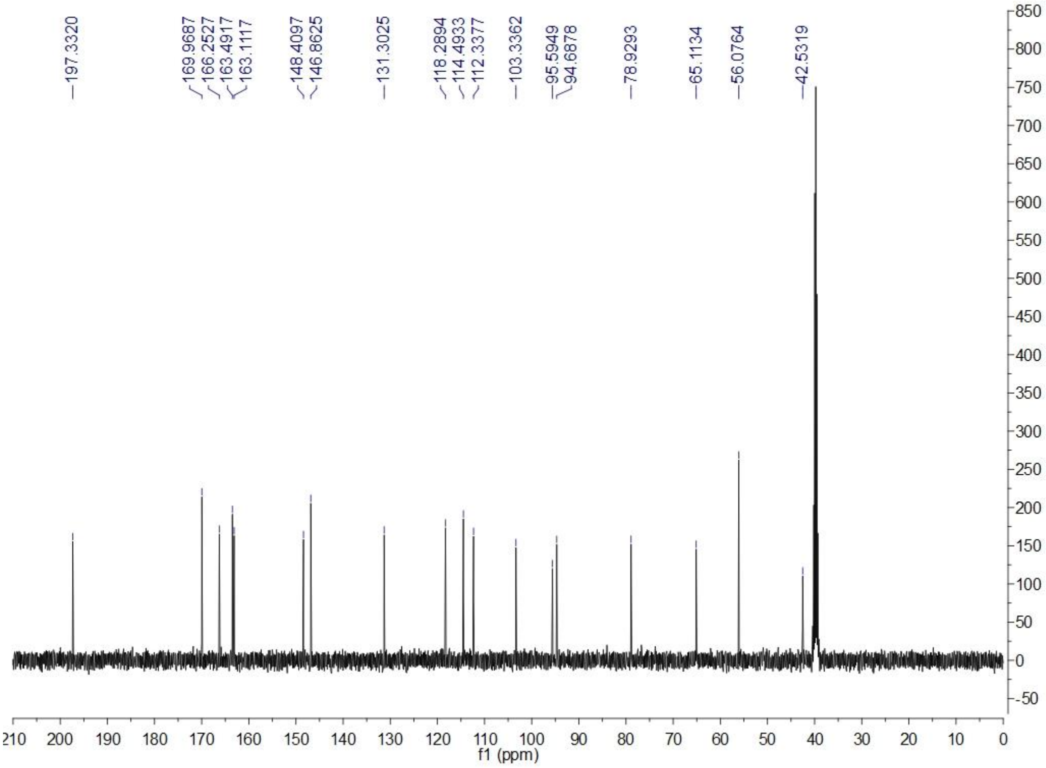

Figure S4. HRMS, ^{1}H NMR and ^{13}C NMR spectra for the target compound **4a:**

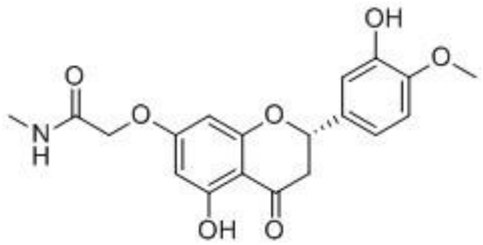

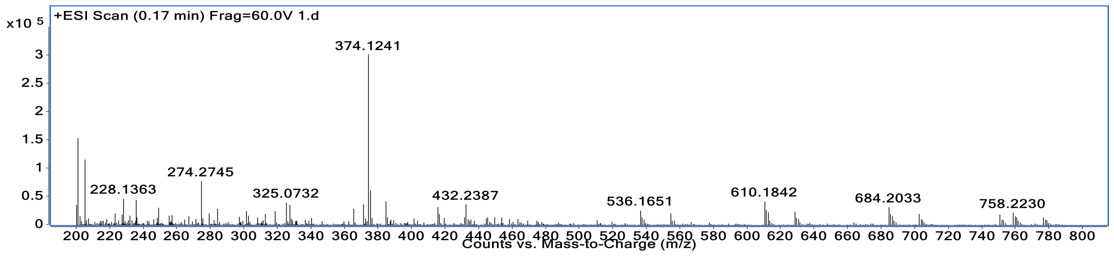

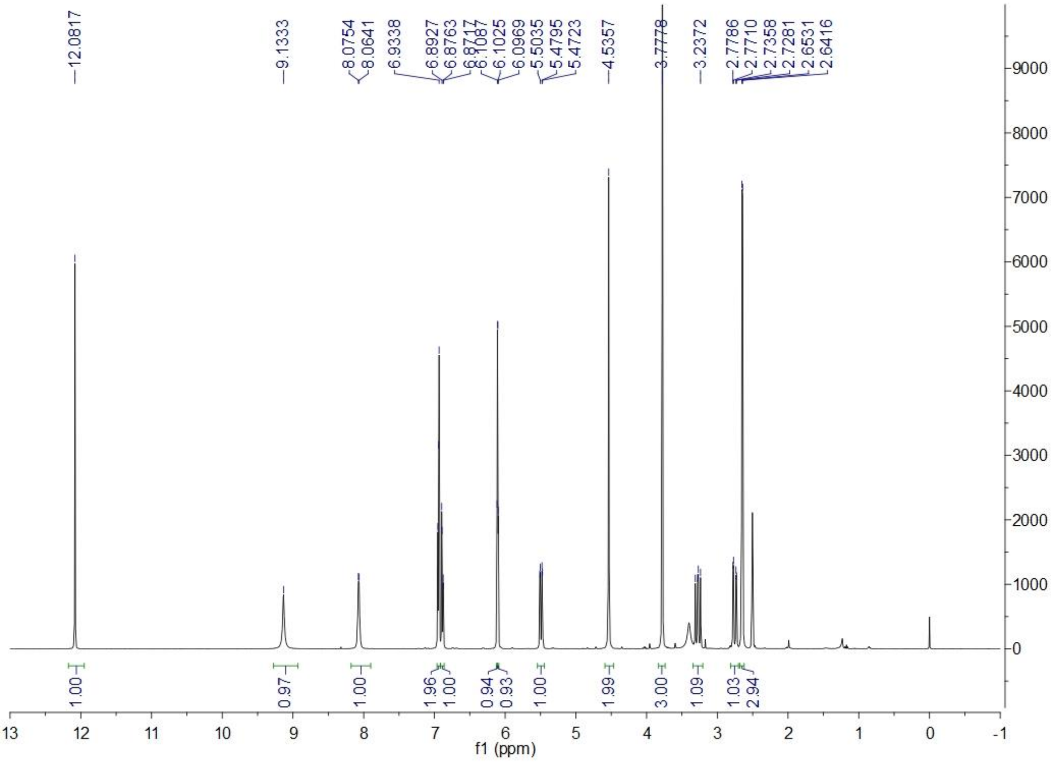

12.0817
9.1333
8.0754
8.0641
6.9338
6.8927
6.8763
6.8717
6.1087
6.1025
6.0969
5.5035
5.4795
5.4723
4.5357
3.7778
3.2372
2.7786
2.7710
2.7358
2.7281
2.6531
2.6416
1.00
0.97
1.00
1.96
1.00
0.94
0.93
1.00
1.99
3.00
1.09
1.03
2.94
f1 (ppm)

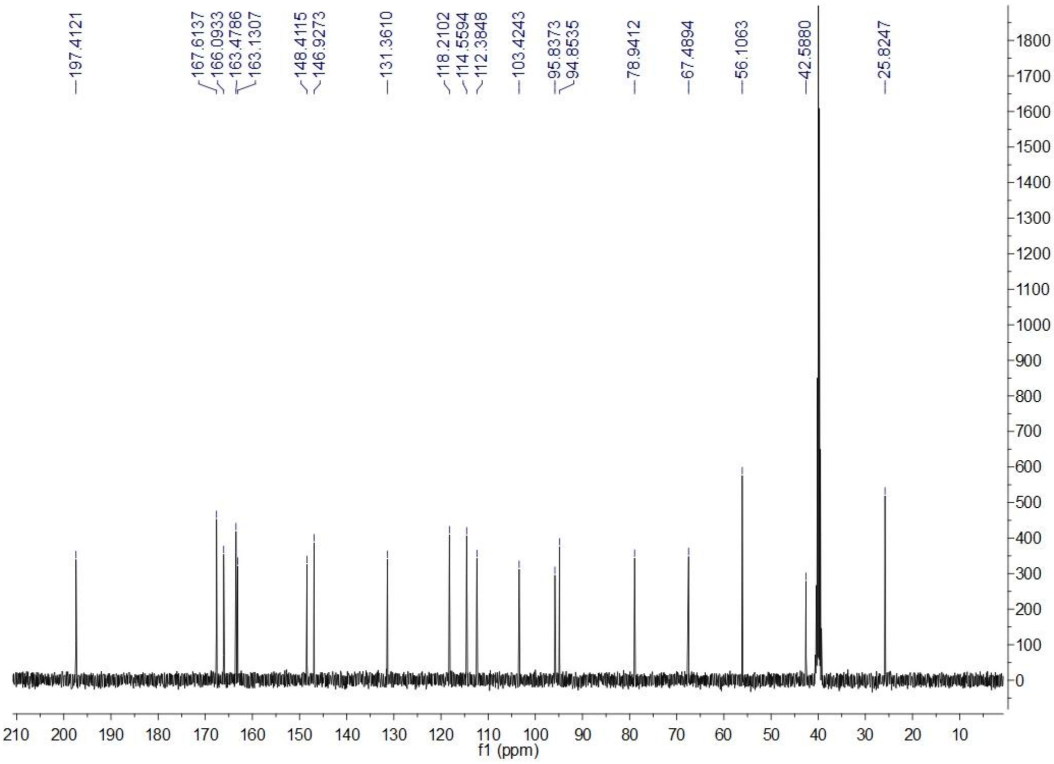

197.4121
167.6137
166.0933
163.4786
163.1307
148.4115
146.9273
131.3610
118.2102
114.5594
112.3848
103.4243
95.8373
94.8535
78.9412
67.4894
56.1063
42.5880
25.8247
f1 (ppm)

Figure S5. HRMS, ^{1}H NMR and ^{13}C NMR spectra for the target compound **4b:**

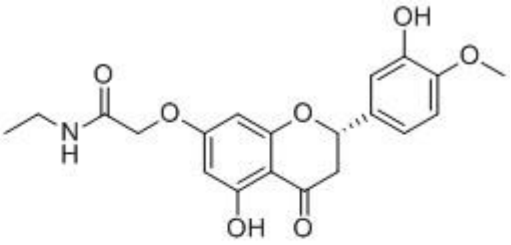

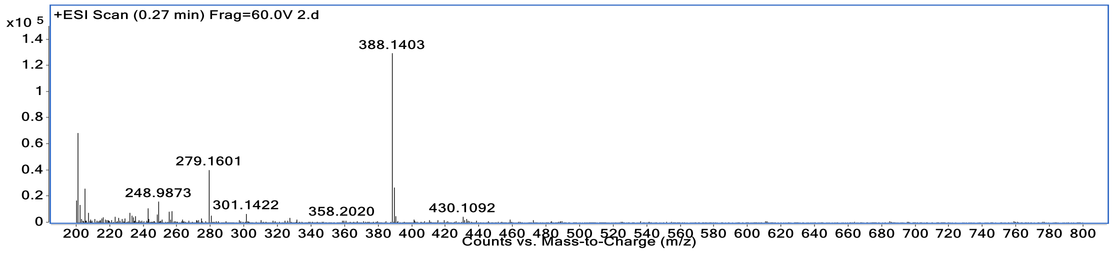

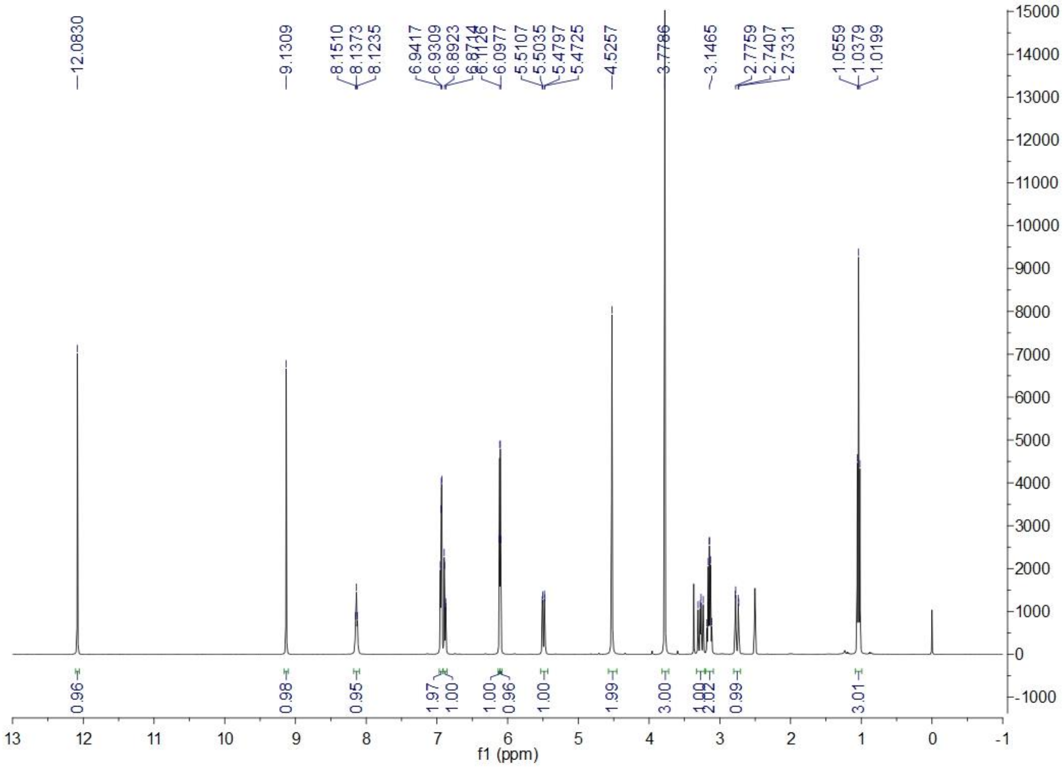

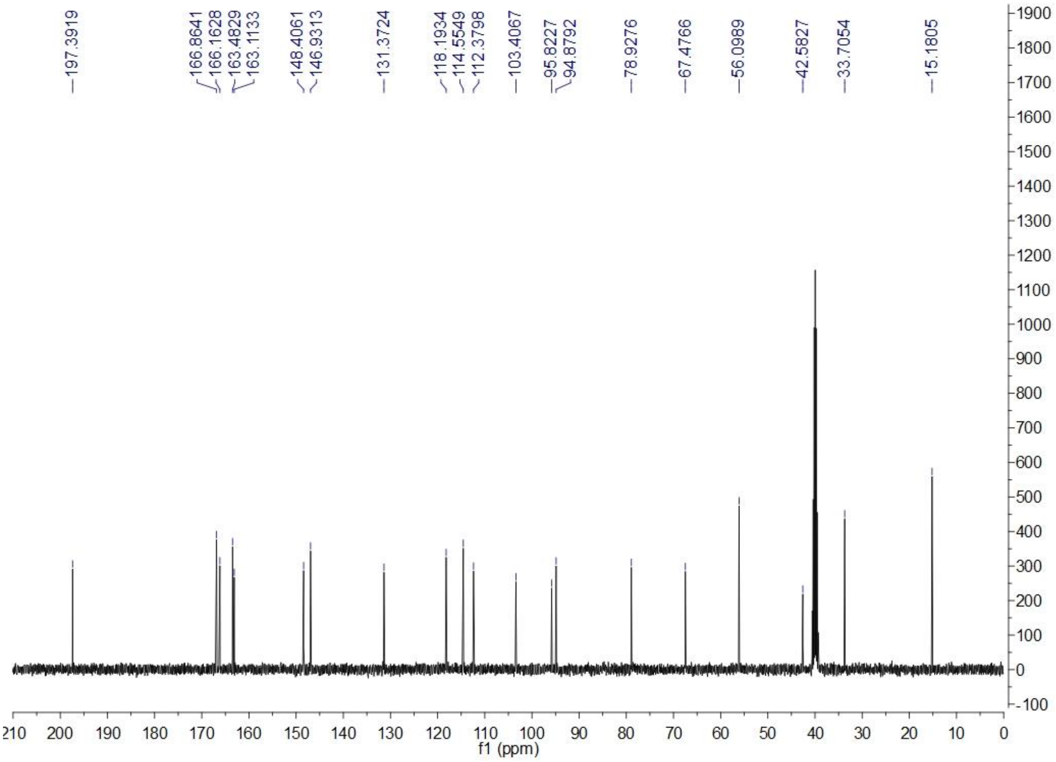

Figure S6. HRMS, ^{1}H NMR and ^{13}C NMR spectra for the target compound **4c:**

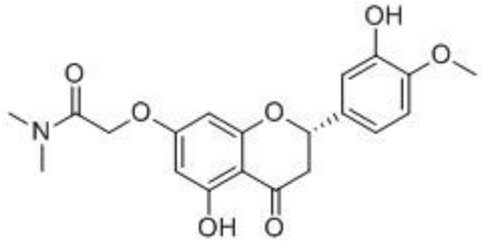

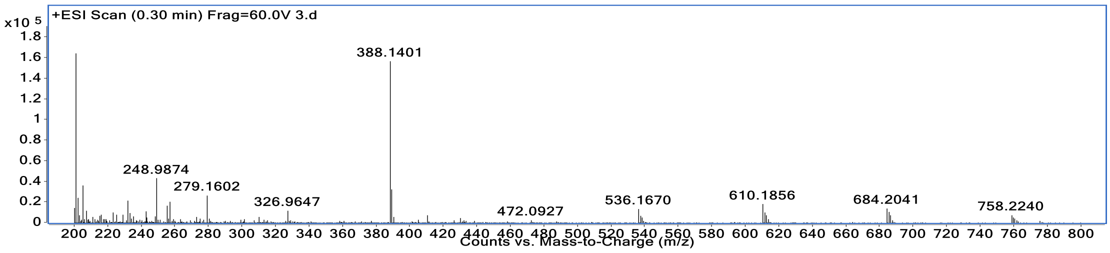

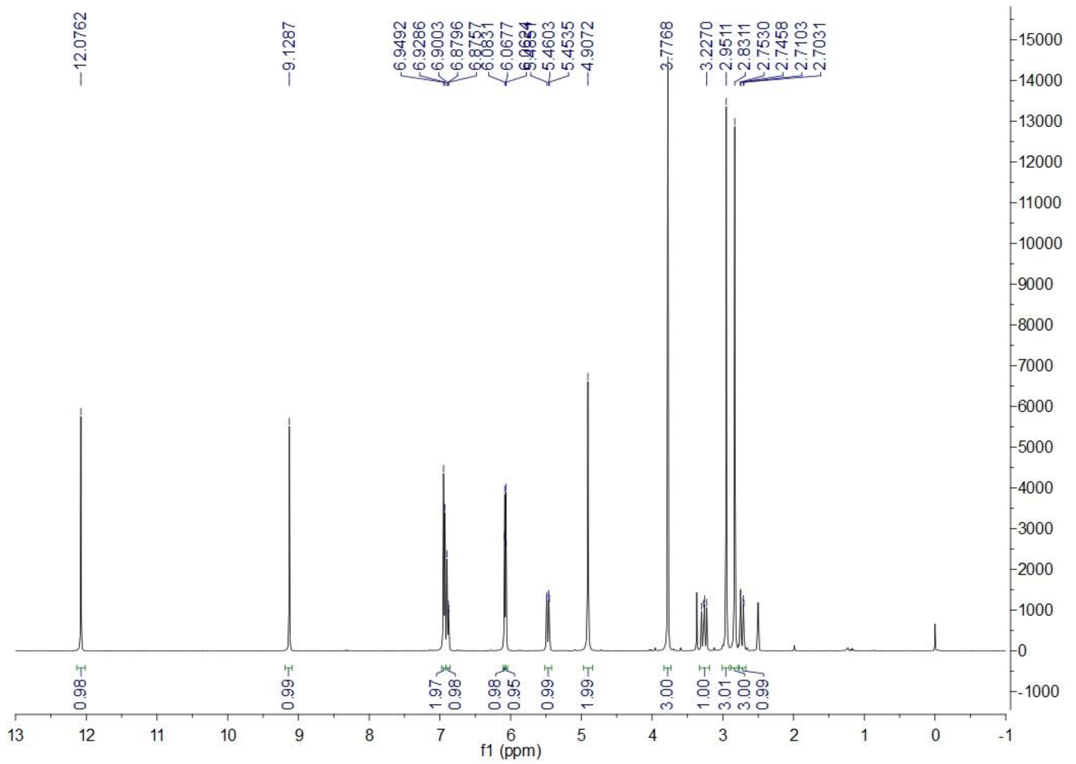

12.0762
9.1287
6.9492
6.9286
6.9003
6.8796
6.8757
6.0831
6.0677
5.4603
5.4535
4.9072
3.7768
3.2270
2.9511
2.8311
2.7530
2.7458
2.7103
2.7031
0.98
0.99
1.97
0.98
0.98
0.95
0.99
1.99
3.00
1.00
3.01
3.00
0.99
f1 (ppm)

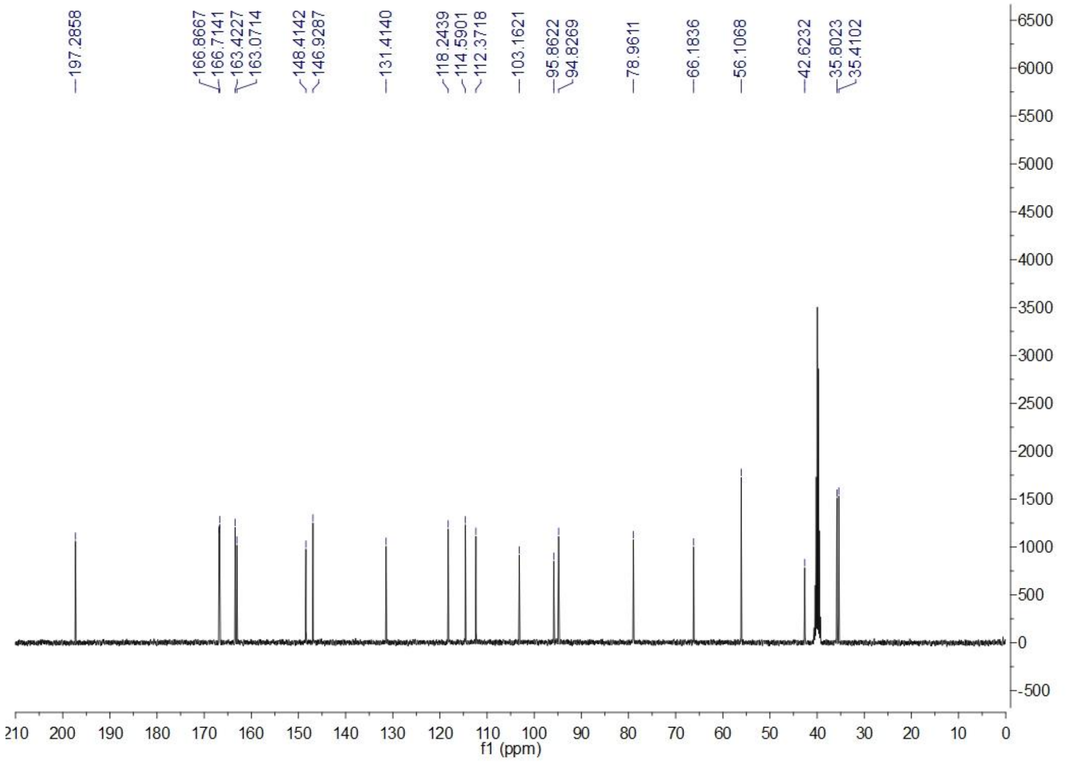

197.2858
166.8667
166.7141
163.4227
163.0714
148.4142
146.9287
131.4140
118.2439
114.5901
112.3718
103.1621
95.8622
94.8269
78.9611
66.1836
56.1068
42.6232
35.8023
35.4102
f1 (ppm)

Figure S7. HRMS, ^{1}H NMR and ^{13}C NMR spectra for the target compound **4d:**

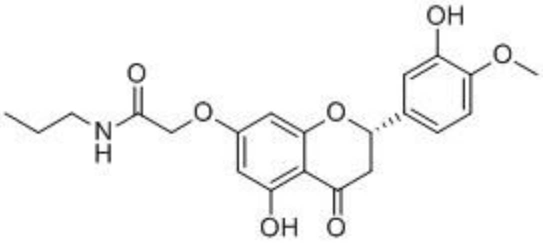

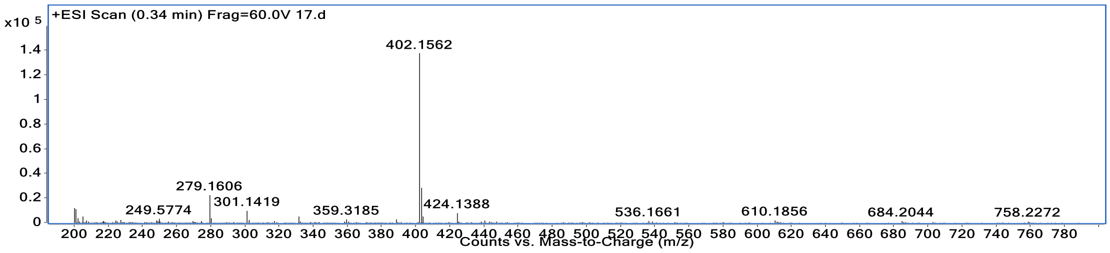

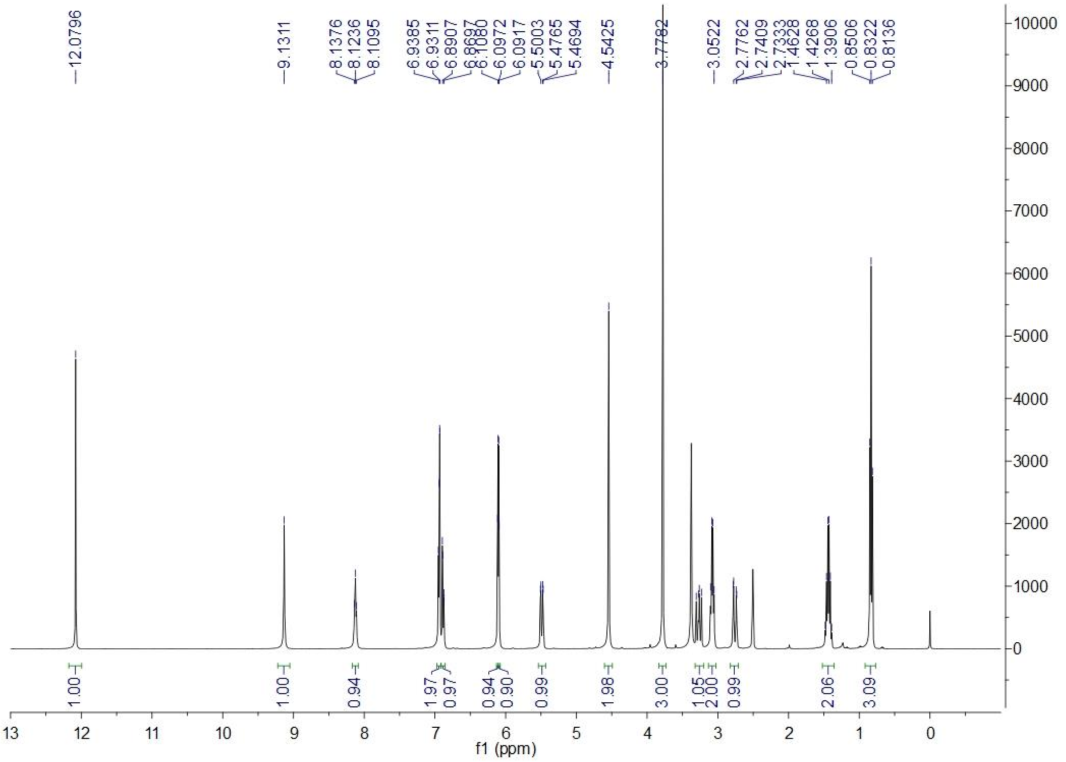

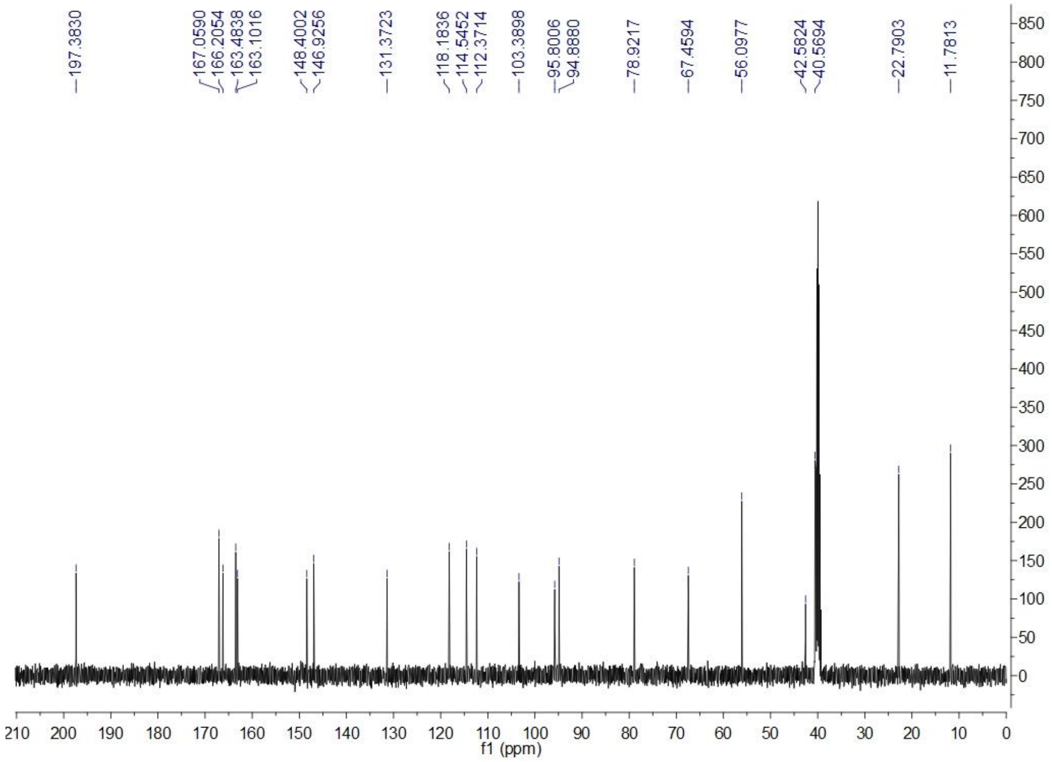

Figure S8. HRMS, ^{1}H NMR and ^{13}C NMR spectra for the target compound **4e:**

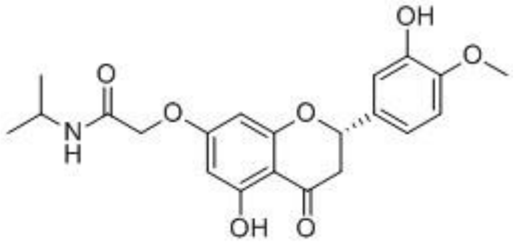

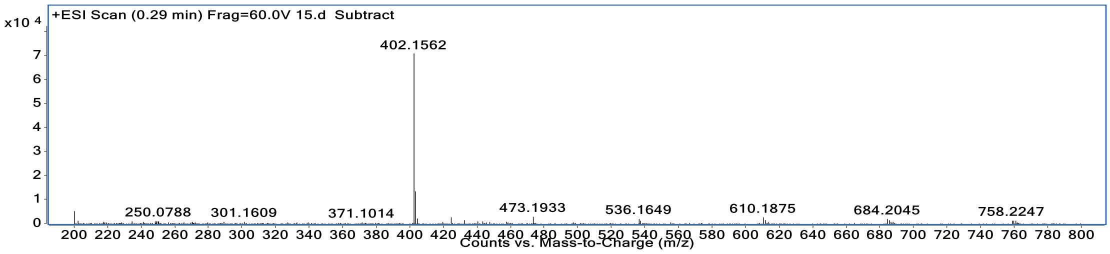

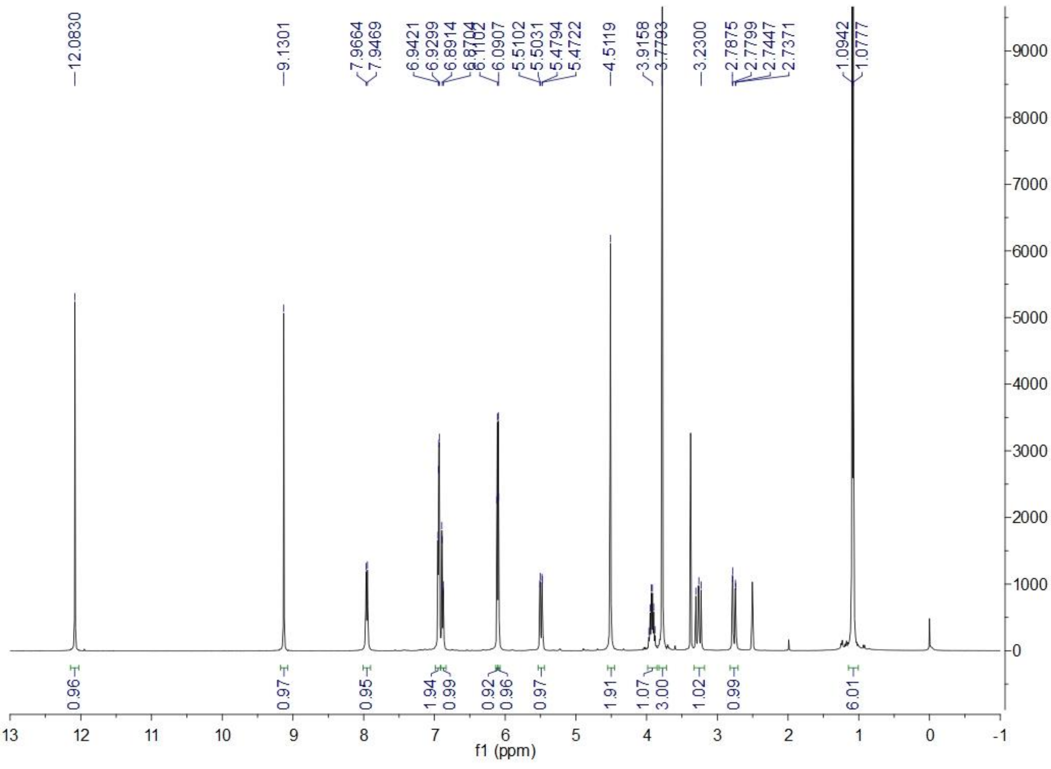
12.0830
9.1301
7.9664
7.9469
6.9421
6.9299
6.8914
6.0907
5.5102
5.5031
5.4794
5.4722
4.5119
3.9158
3.2300
2.7875
2.7799
2.7447
2.7371
1.0942
1.0777
0.96
0.97
0.95
1.94
0.99
0.92
0.96
0.97
1.91
1.07
3.00
1.02
0.99
6.01
f1 (ppm)

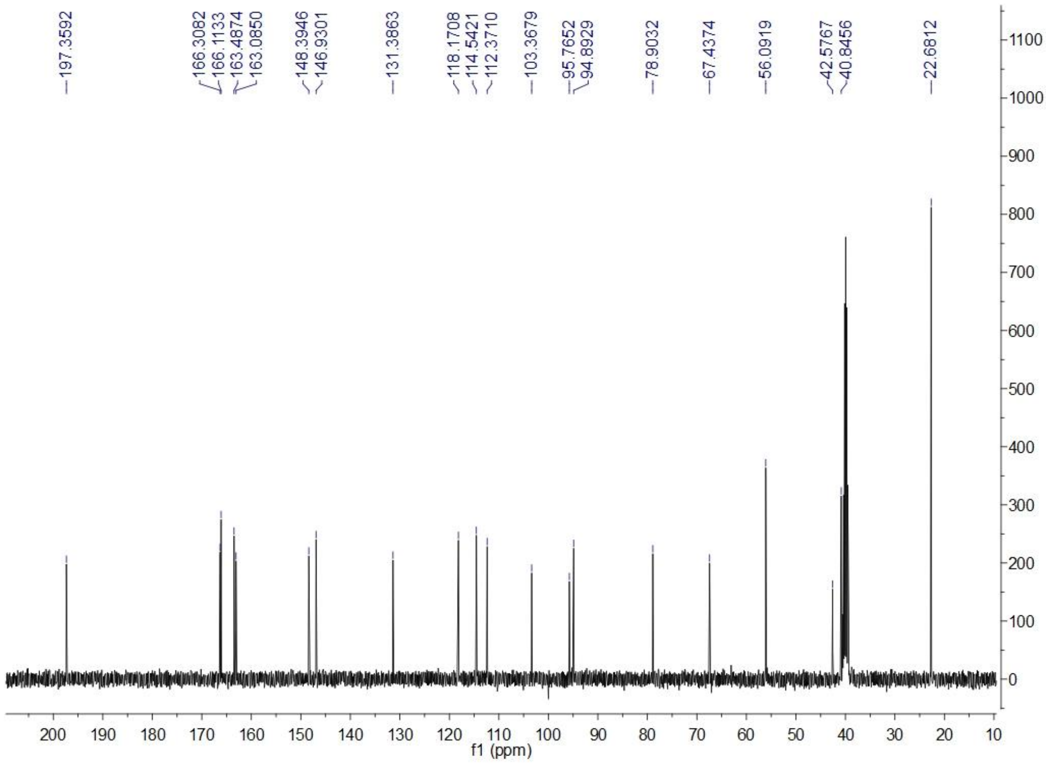
197.3592
166.3082
166.1133
163.4874
163.0850
148.3946
146.9301
131.3863
118.1708
114.5421
112.3710
103.3679
95.7652
94.8929
78.9032
67.4374
56.0919
42.5767
40.8456
22.6812
f1 (ppm)

Figure S9. HRMS, ^{1}H NMR and ^{13}C NMR spectra for the target compound **4f:**

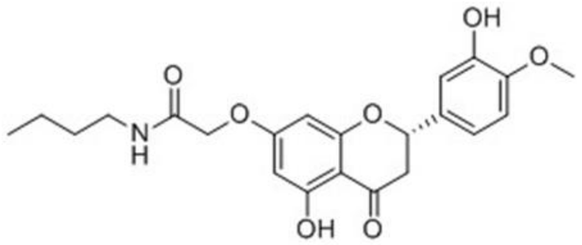

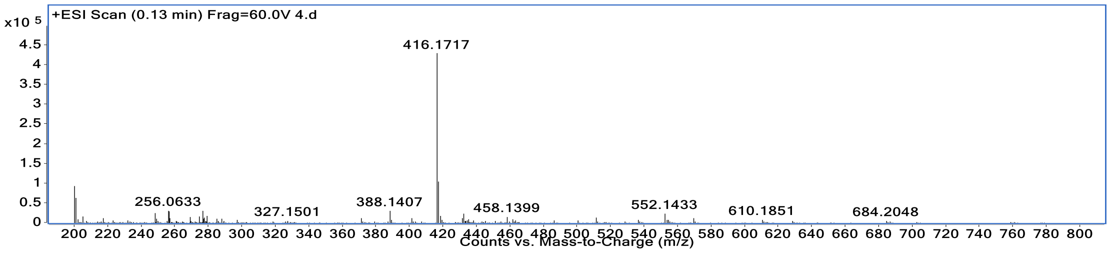

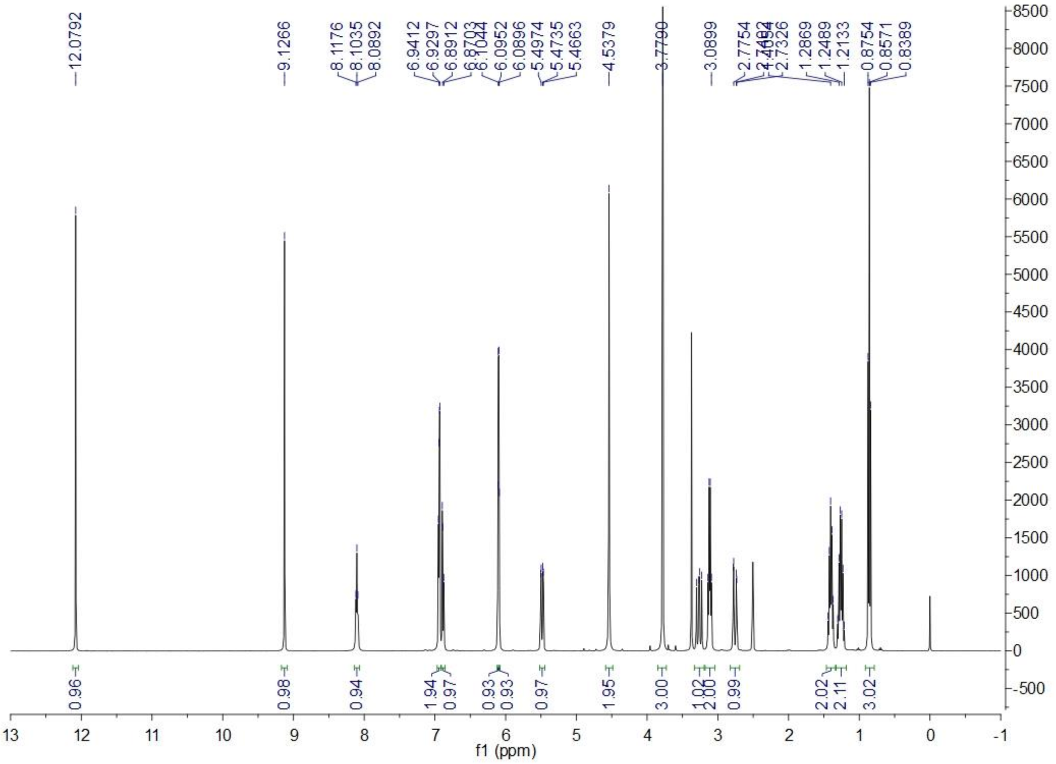

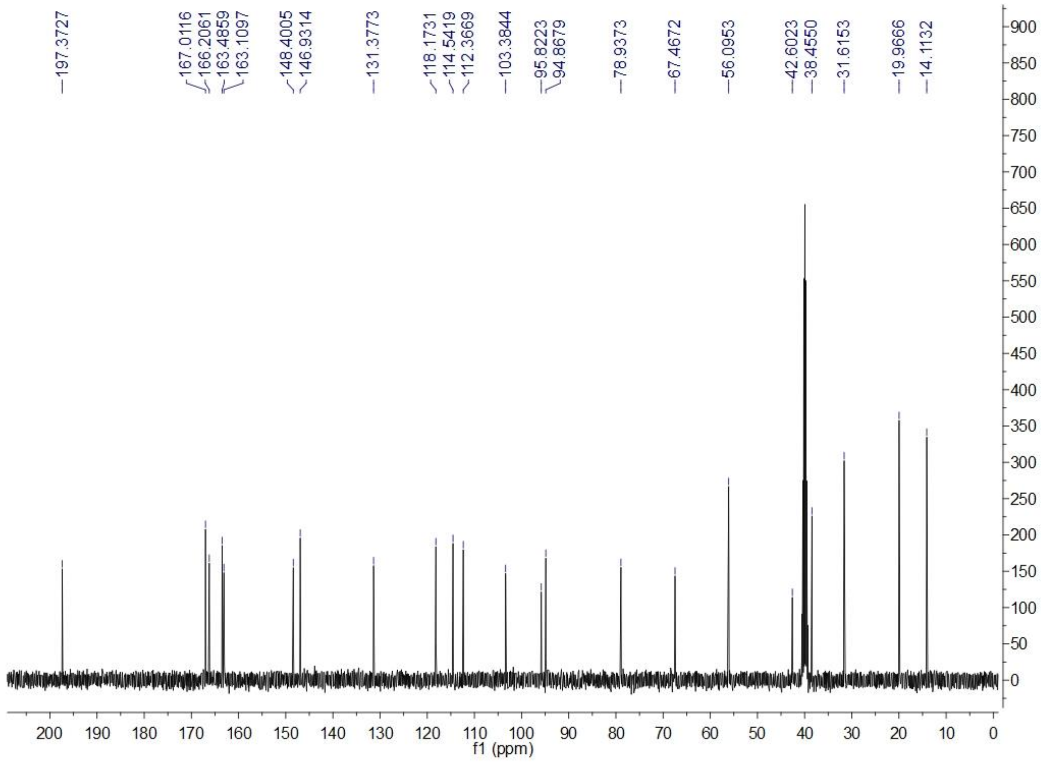

Figure S10. HRMS, ^{1}H NMR and ^{13}C NMR spectra for the target compound **4g:**

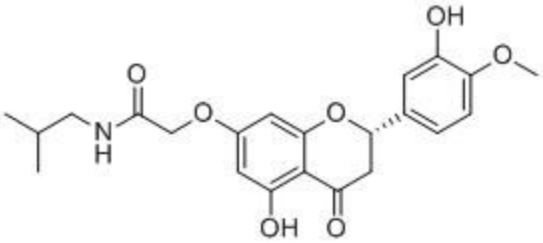

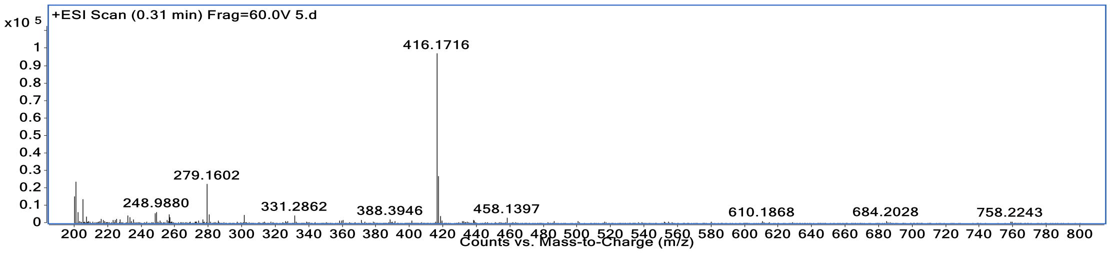

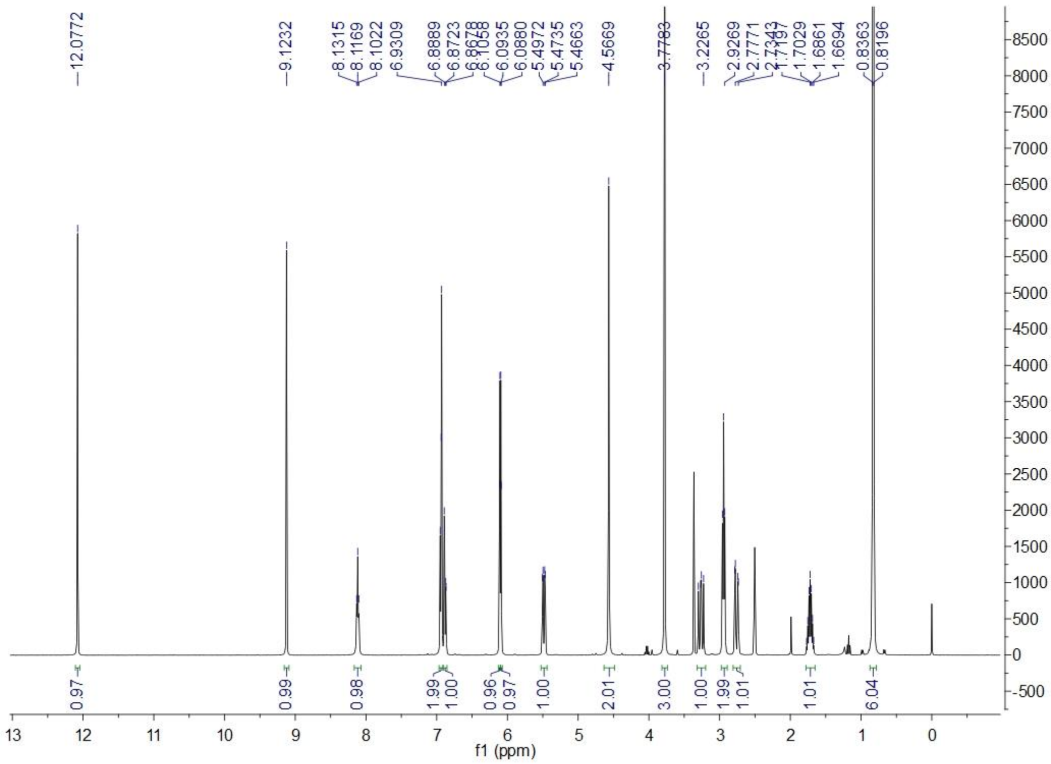
12.0772
9.1232
8.1315
8.1169
8.1022
6.9309
6.8889
6.8723
6.0935
6.0880
5.4972
5.4735
5.4663
4.5669
3.7783
3.2265
2.9269
2.7771
1.7029
1.6861
1.6694
0.8363
0.8196
0.97
0.99
0.98
1.99
1.00
0.96
0.97
1.00
2.01
3.00
1.00
1.99
1.01
1.01
6.04
f1 (ppm)
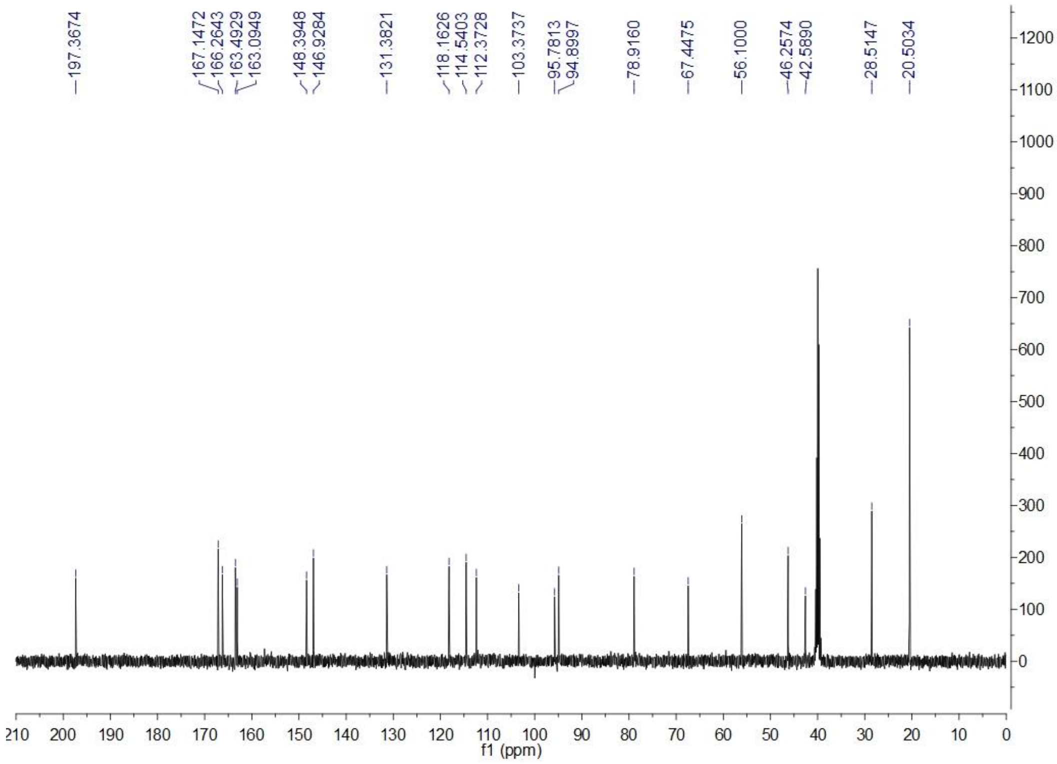
197.3674
167.1472
166.2643
163.4929
163.0949
148.3948
146.9284
131.3821
118.1626
114.5403
112.3728
103.3737
95.7813
94.8997
78.9160
67.4475
56.1000
46.2574
42.5890
28.5147
20.5034
f1 (ppm)

Figure S11. HRMS, ^{1}H NMR and ^{13}C NMR spectra for the target compound **4h:**

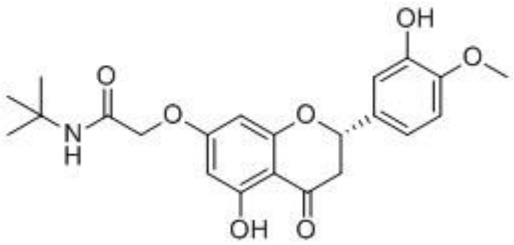

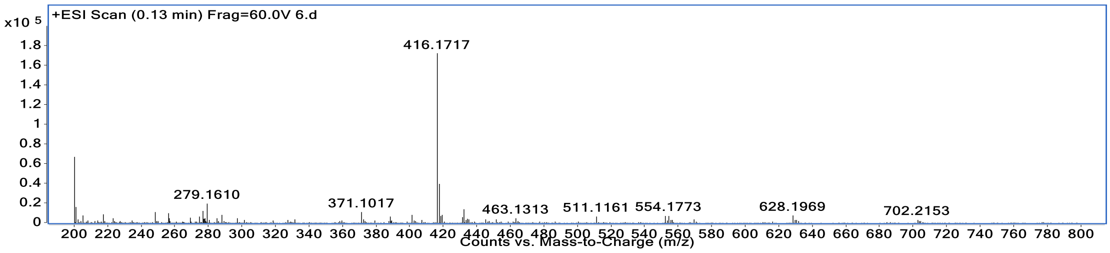

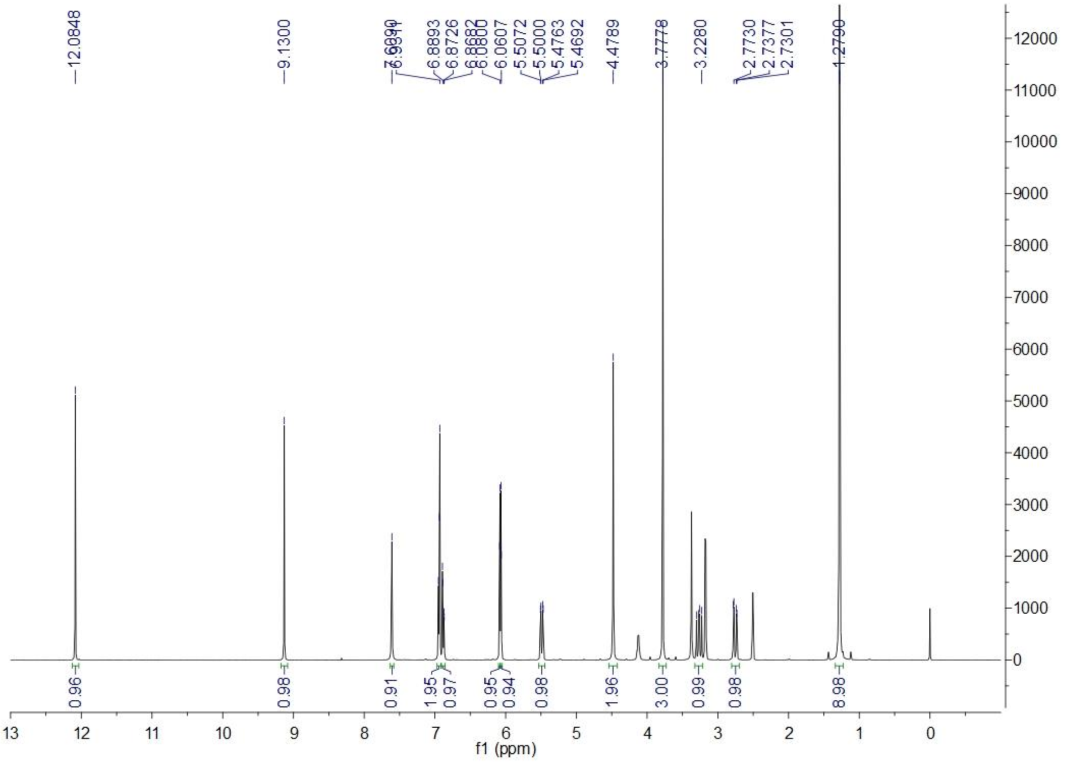

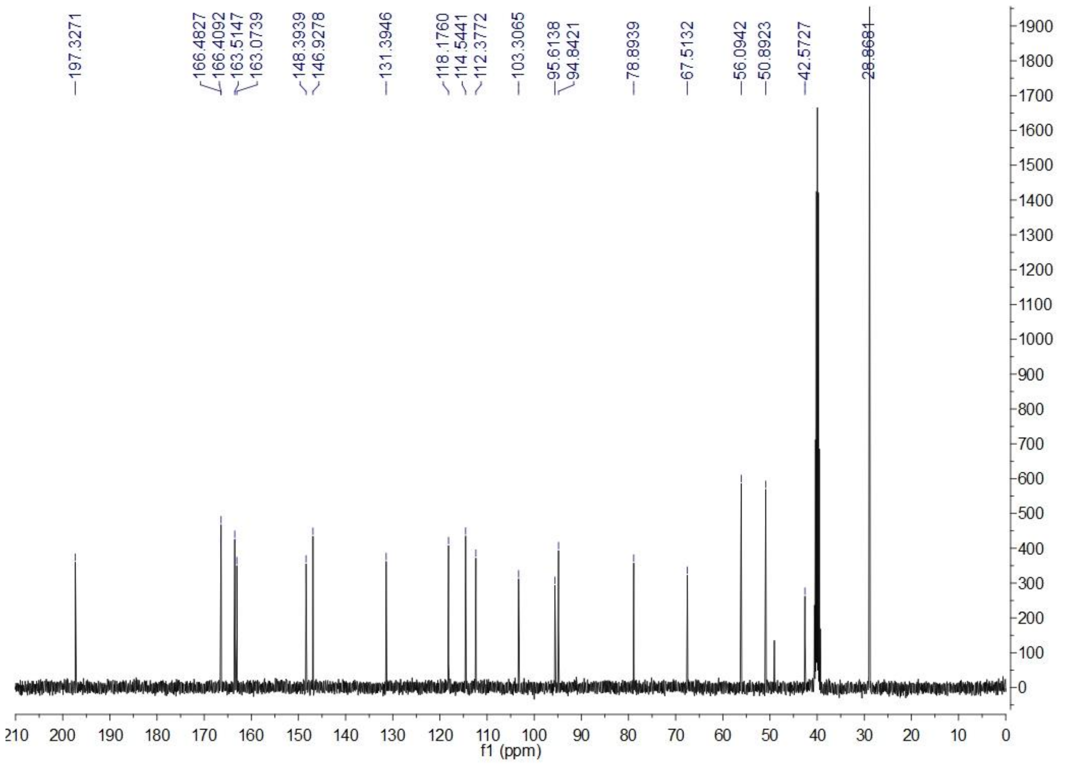

Figure S12. HRMS, ^{1}H NMR and ^{13}C NMR spectra for the target compound **4i:**

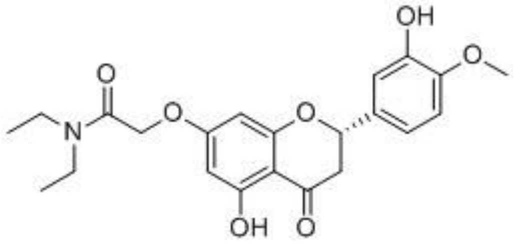

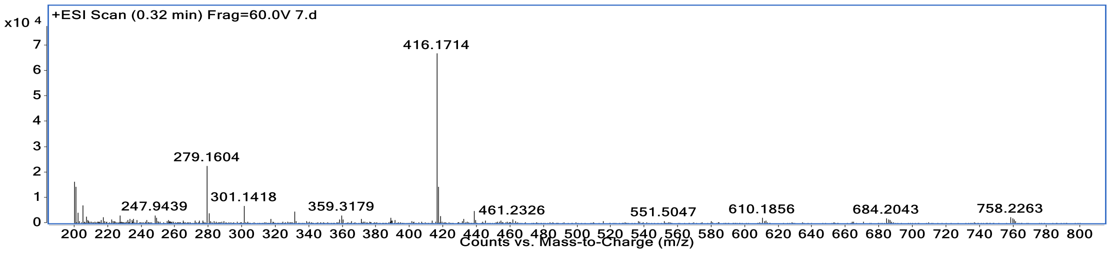

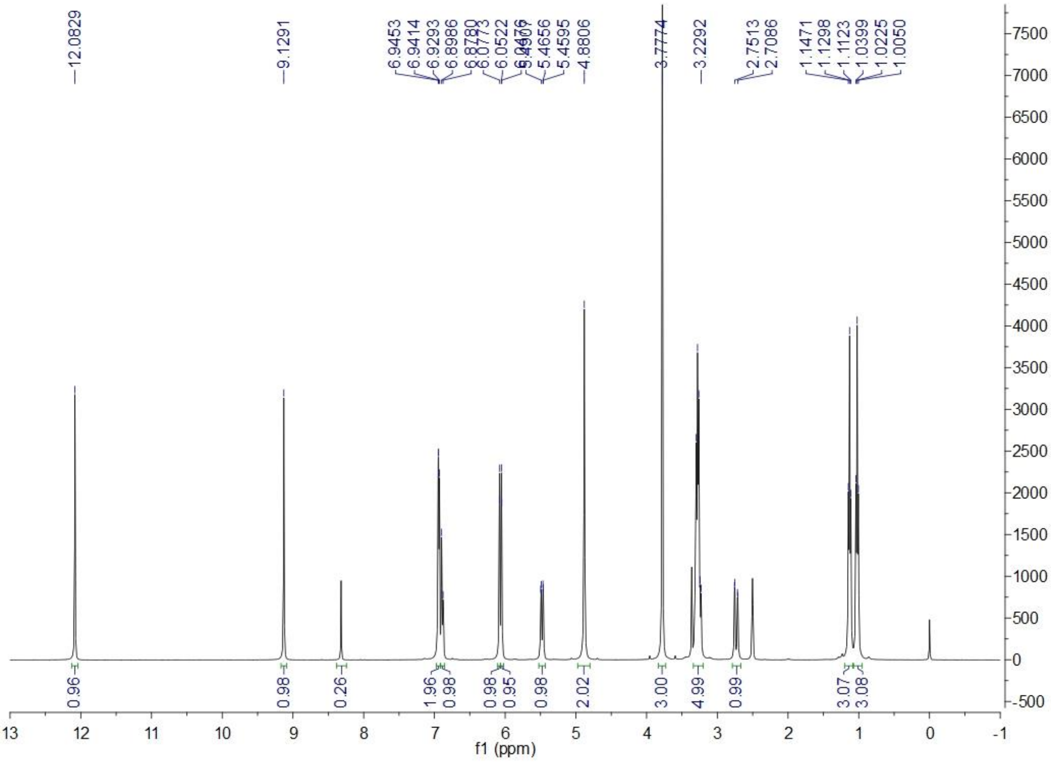
12.0829
9.1291
6.9453
6.9414
6.9293
6.8986
6.0522
5.4656
5.4595
4.8806
3.7774
3.2292
2.7513
2.7086
1.1471
1.1298
1.1123
1.0399
1.0225
1.0050
0.96
0.98
0.26
1.96
0.98
0.98
0.95
0.98
2.02
3.00
4.99
0.99
3.07
3.08
f1 (ppm)

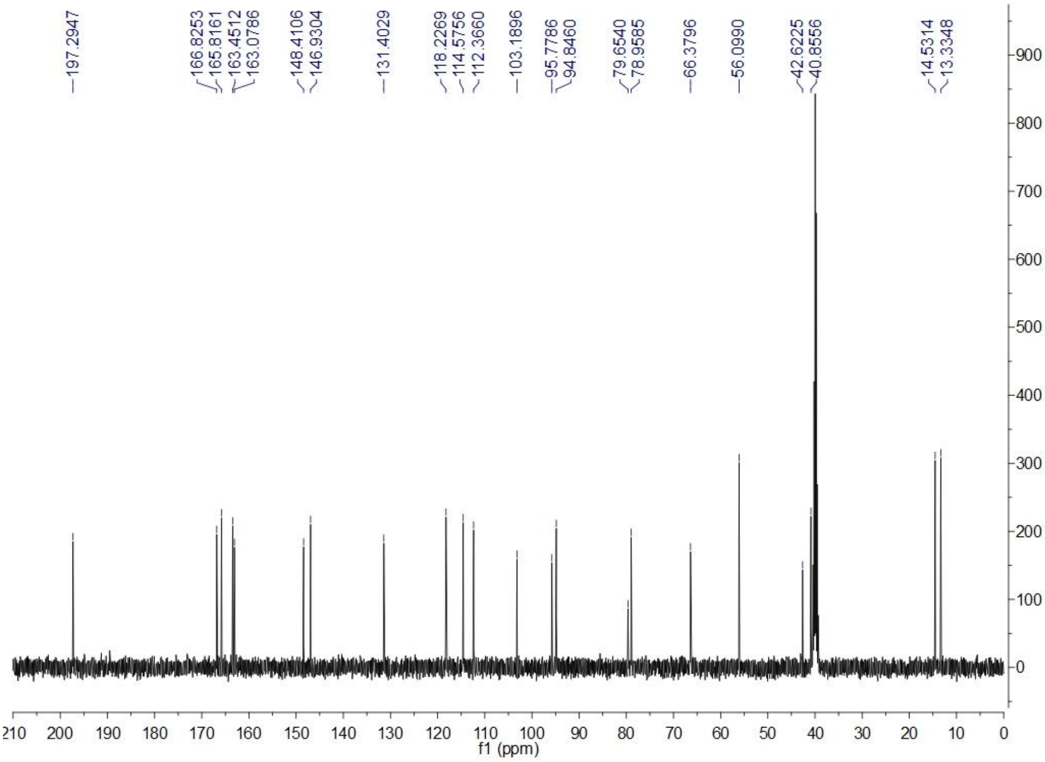
197.2947
166.8253
165.8161
163.4512
163.0786
148.4106
146.9304
131.4029
118.2269
114.5756
112.3660
103.1896
95.7786
94.8460
79.6540
78.9585
66.3796
56.0990
42.6225
40.8556
14.5314
13.3348
f1 (ppm)

Figure S13. HRMS, ^{1}H NMR and ^{13}C NMR spectra for the target compound **4j:**

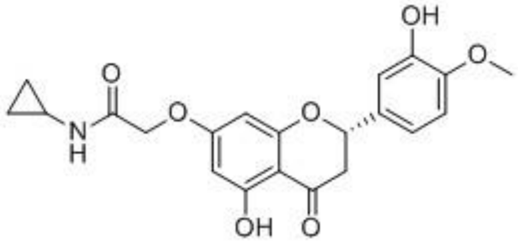

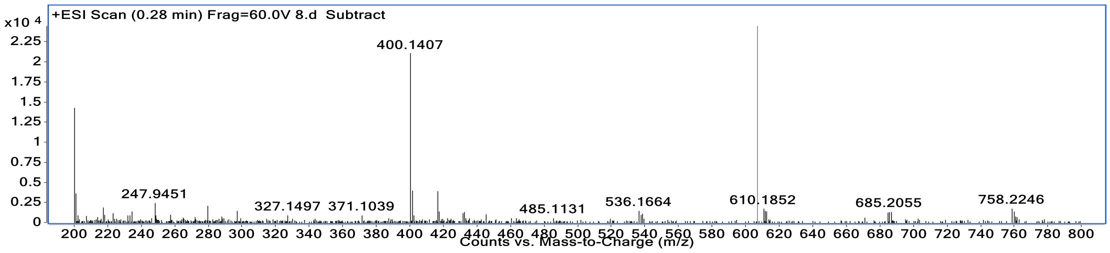

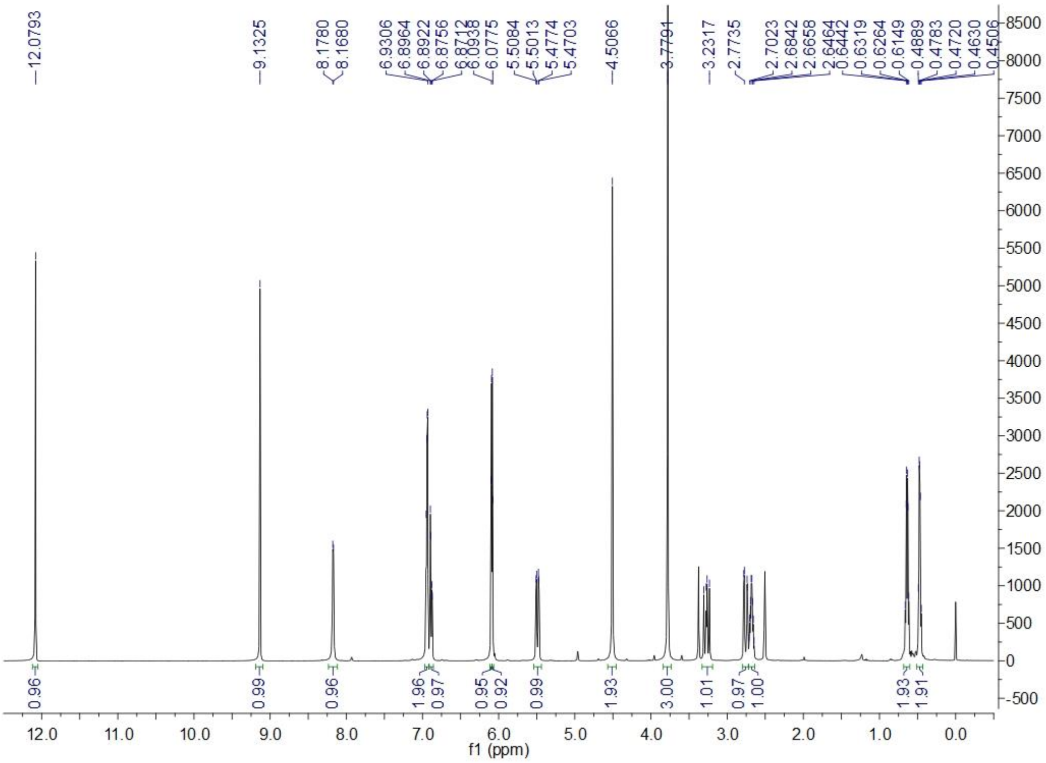

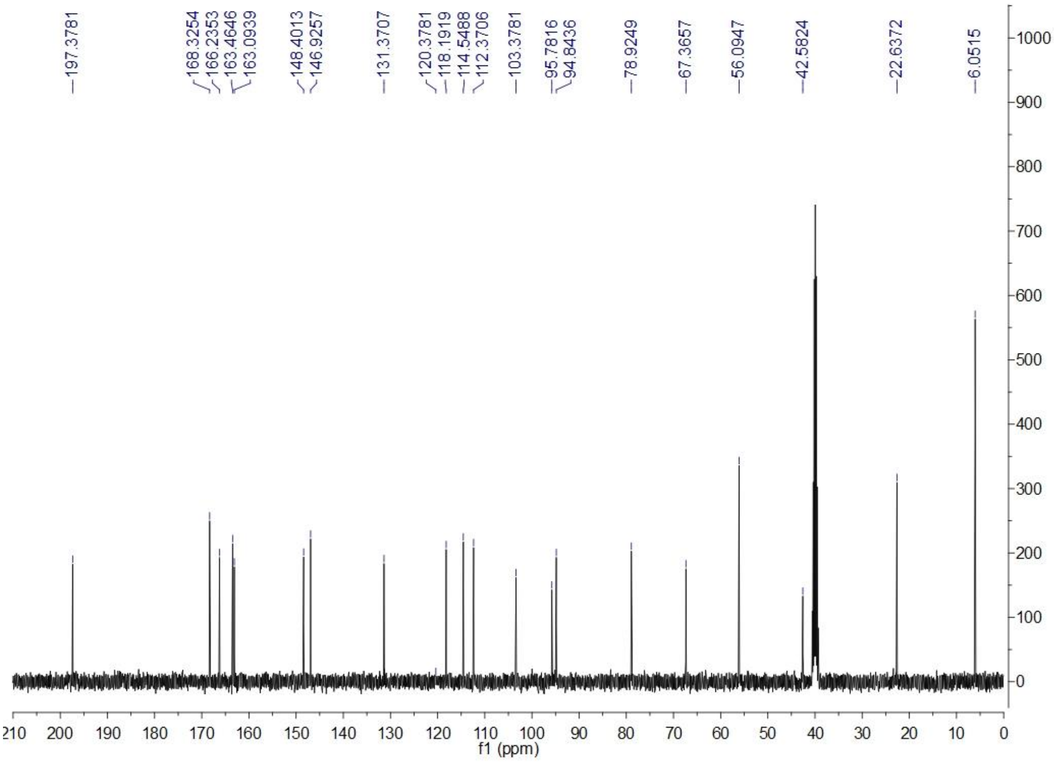

Figure S14. HRMS, ^{1}H NMR and ^{13}C NMR spectra for the target compound **4k:**

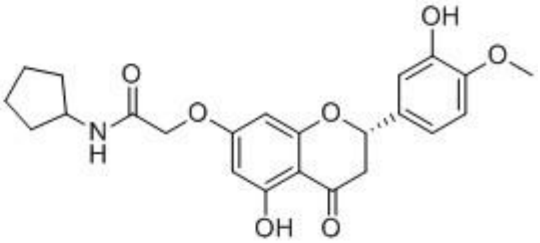

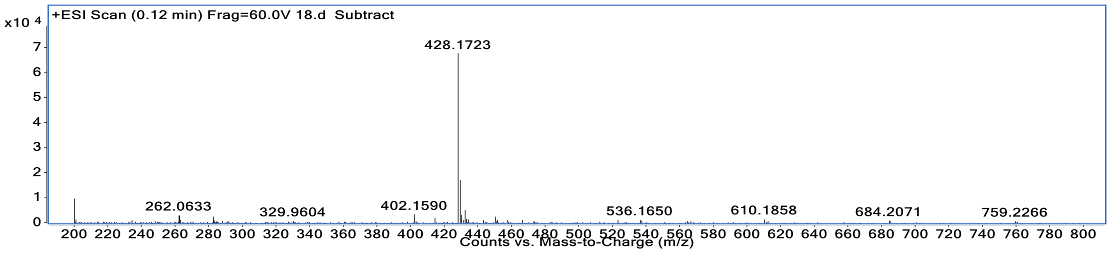

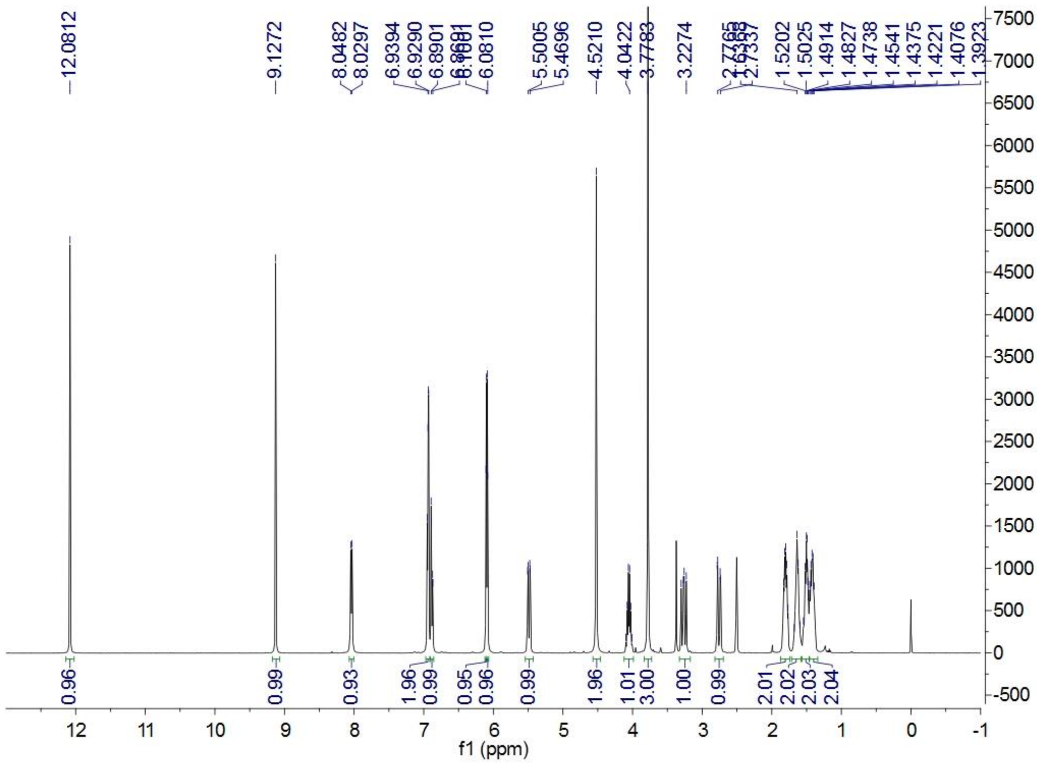

12.0812
9.1272
8.0482
8.0297
6.9394
6.9290
6.8901
6.0810
5.5005
5.4696
4.5210
4.0422
3.7783
3.2274
2.7337
1.5202
1.5025
1.4914
1.4827
1.4738
1.4541
1.4375
1.4221
1.4076
1.3923
0.96
0.99
0.93
1.96
0.99
0.95
0.96
0.99
1.96
1.01
3.00
1.00
0.99
2.01
2.02
2.03
2.04
f1 (ppm)

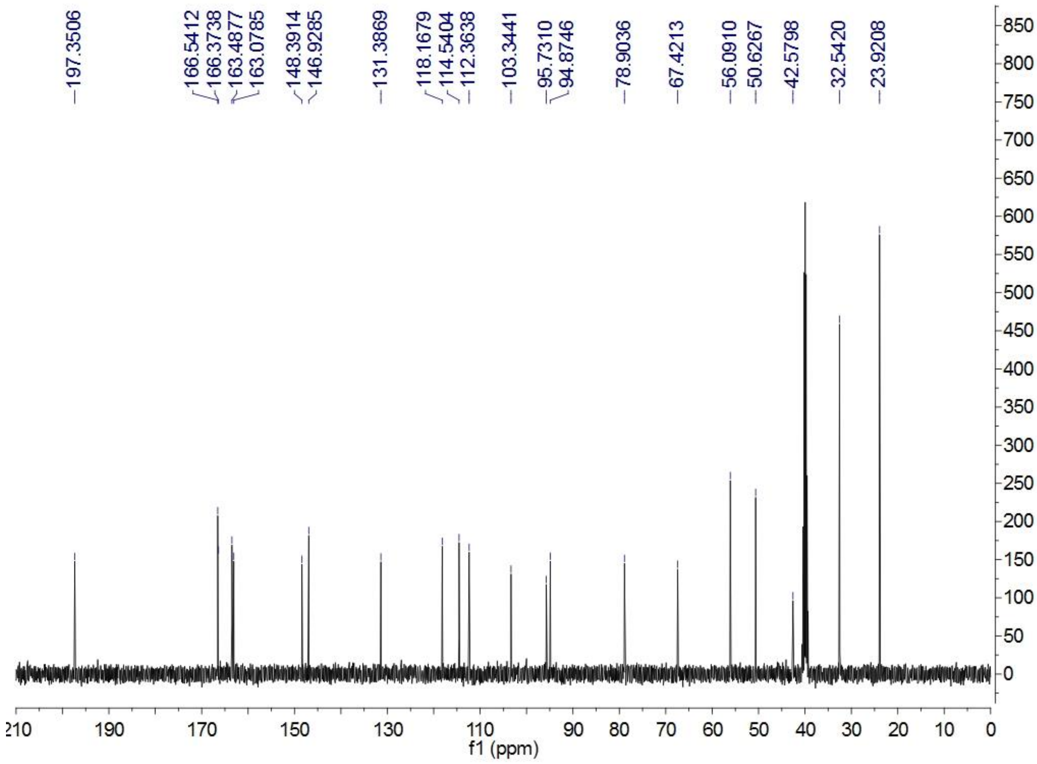

197.3506
166.5412
166.3738
163.4877
163.0785
148.3914
146.9285
131.3869
118.1679
114.5404
112.3638
103.3441
95.7310
94.8746
78.9036
67.4213
56.0910
50.6267
42.5798
32.5420
23.9208
f1 (ppm)

Figure S15. HRMS, ^{1}H NMR and ^{13}C NMR spectra for the target compound **4l:**

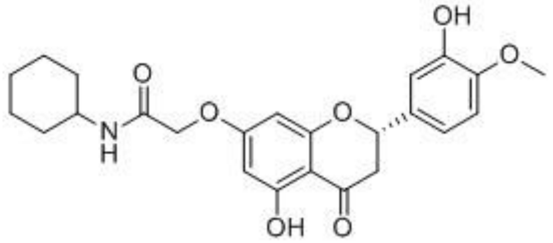

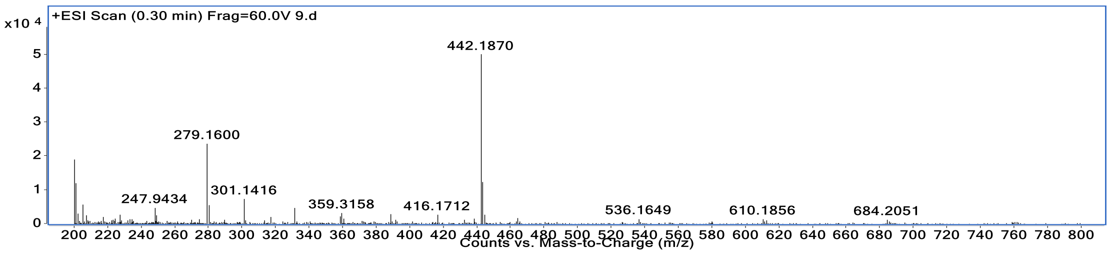

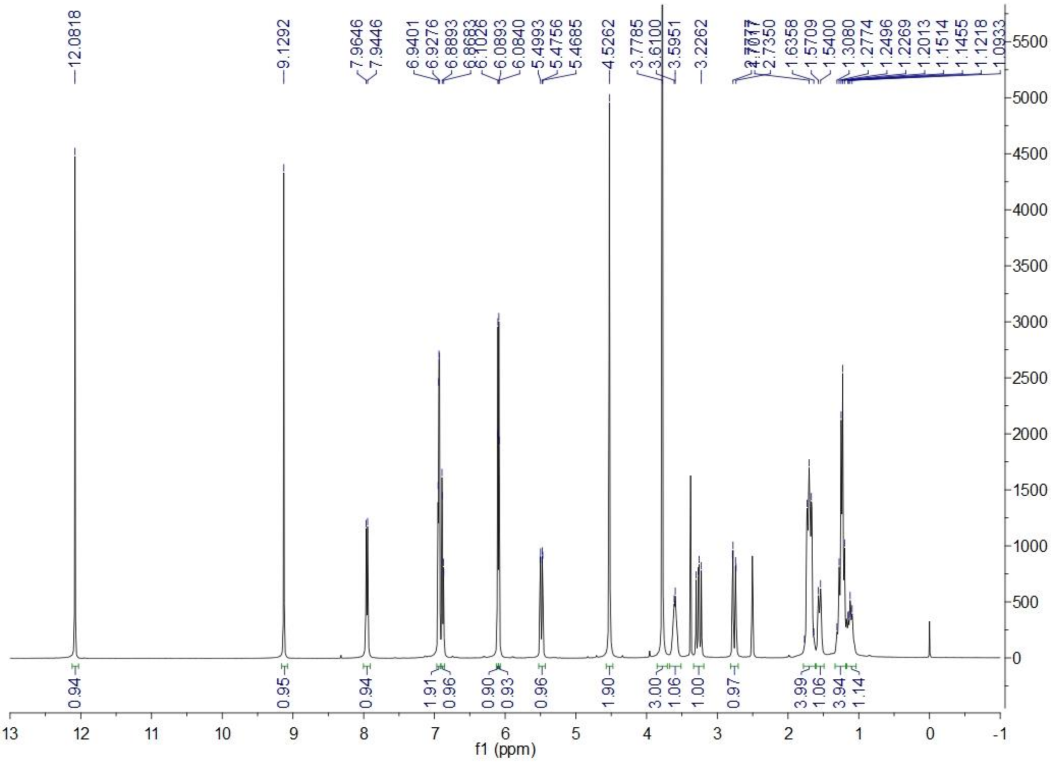

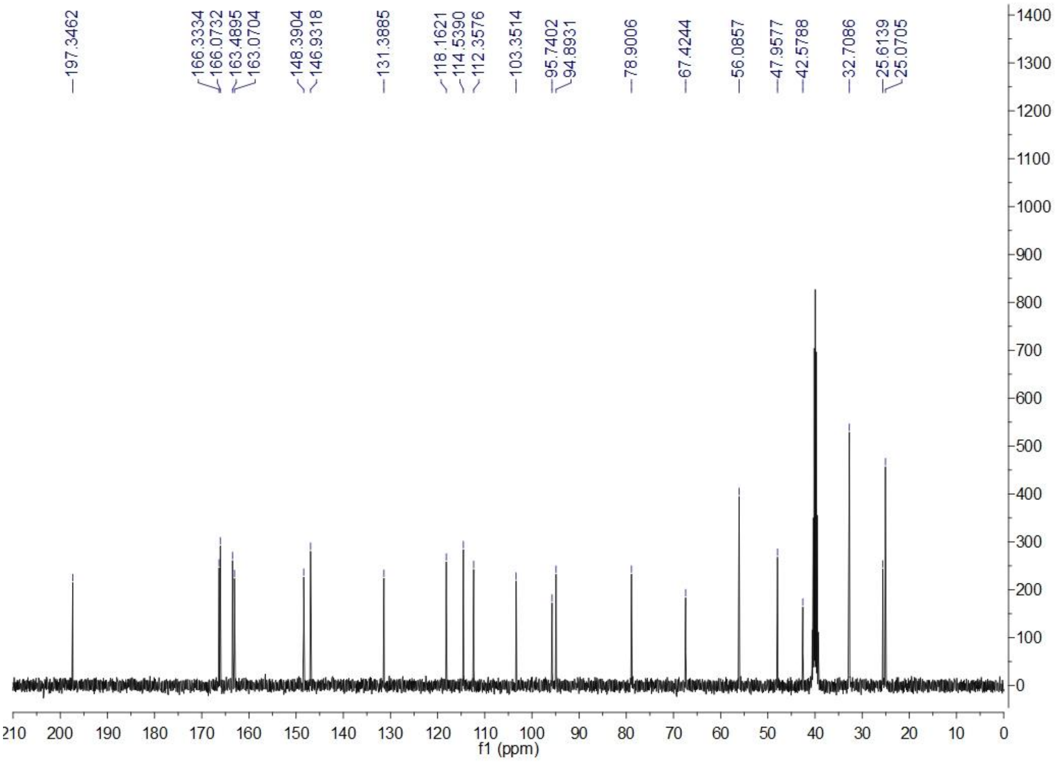

Figure S16. HRMS, ^{1}H NMR and ^{13}C NMR spectra for the target compound **5a:**

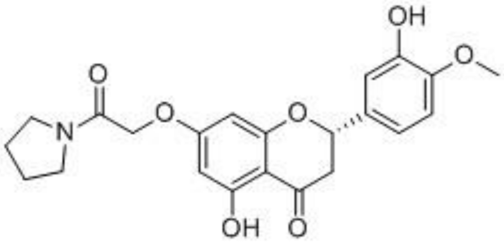

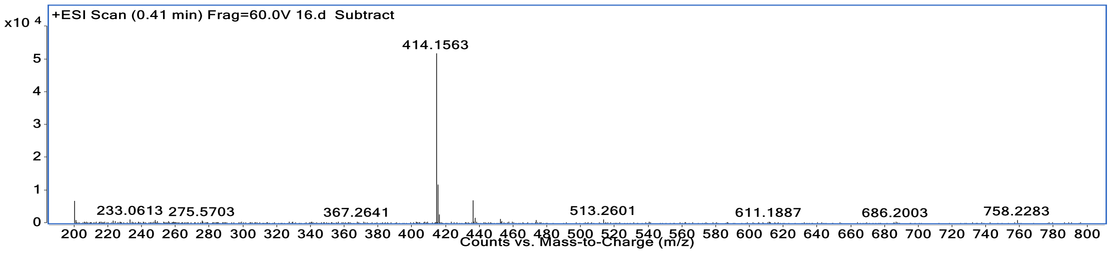

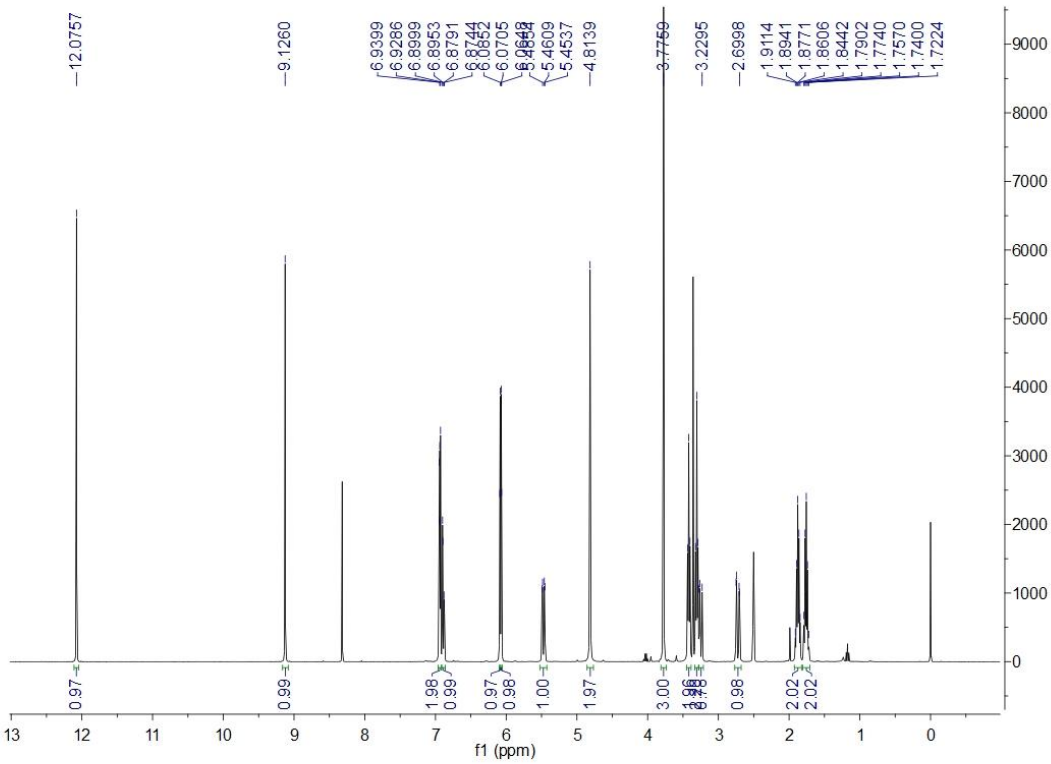
12.0757
9.1260
6.9399
6.9286
6.8999
6.8953
6.8791
6.8744
6.0705
5.4609
5.4537
4.8139
3.7759
3.2295
2.6998
1.9114
1.8941
1.8771
1.8606
1.8442
1.7902
1.7740
1.7570
1.7400
1.7224
0.97
0.99
1.98
0.99
0.97
0.98
1.00
1.97
3.00
0.98
2.02
2.02
f1 (ppm)

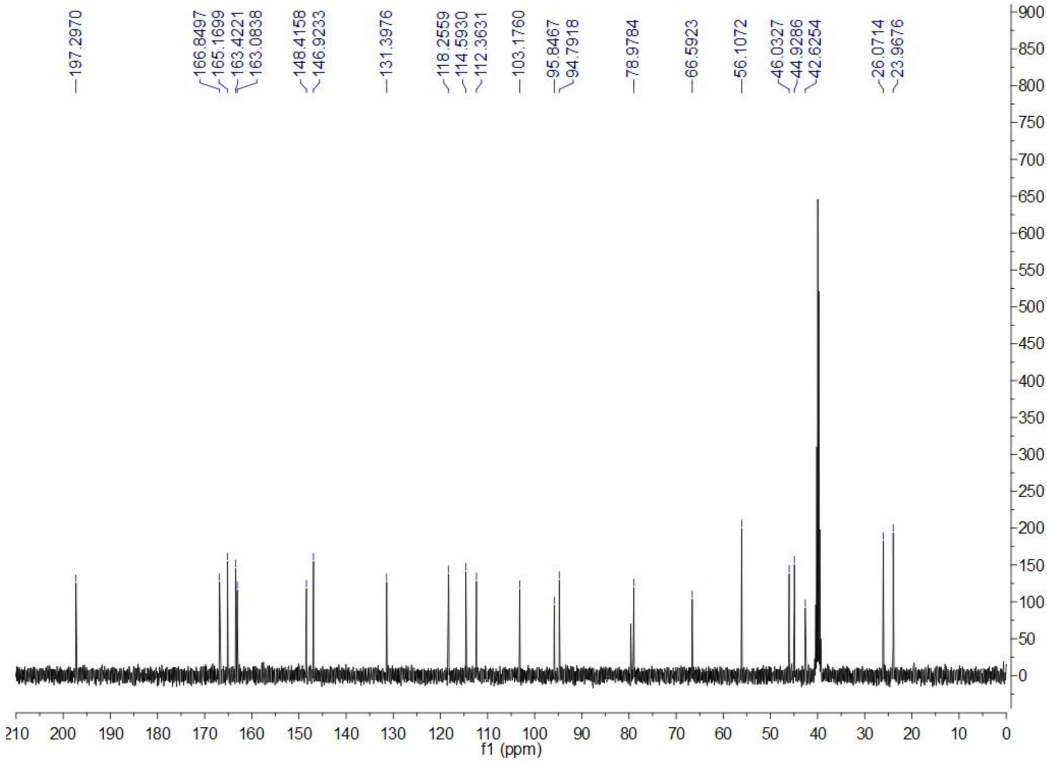
197.2970
166.8497
165.1699
163.4221
163.0838
148.4158
146.9233
131.3976
118.2559
114.5930
112.3631
103.1760
95.8467
94.7918
78.9784
66.5923
56.1072
46.0327
44.9286
42.6254
26.0714
23.9676
f1 (ppm)

Figure S17. HRMS, ^{1}H NMR and ^{13}C NMR spectra for the target compound **5b:**

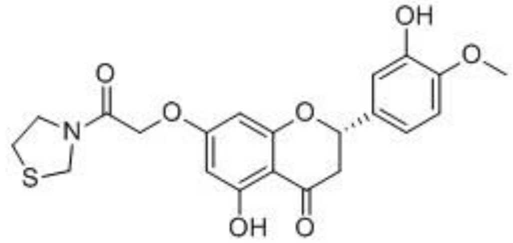

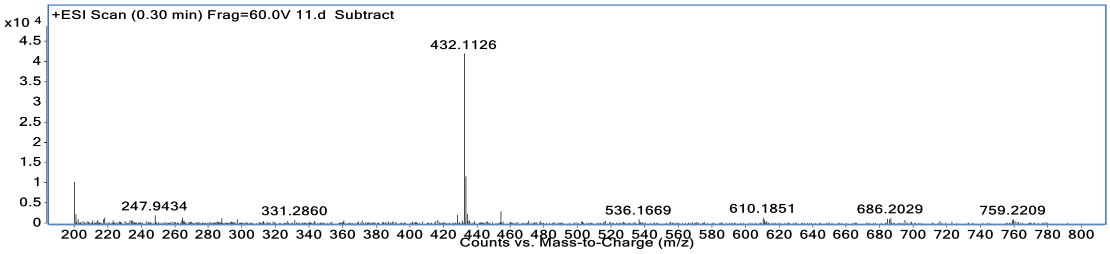

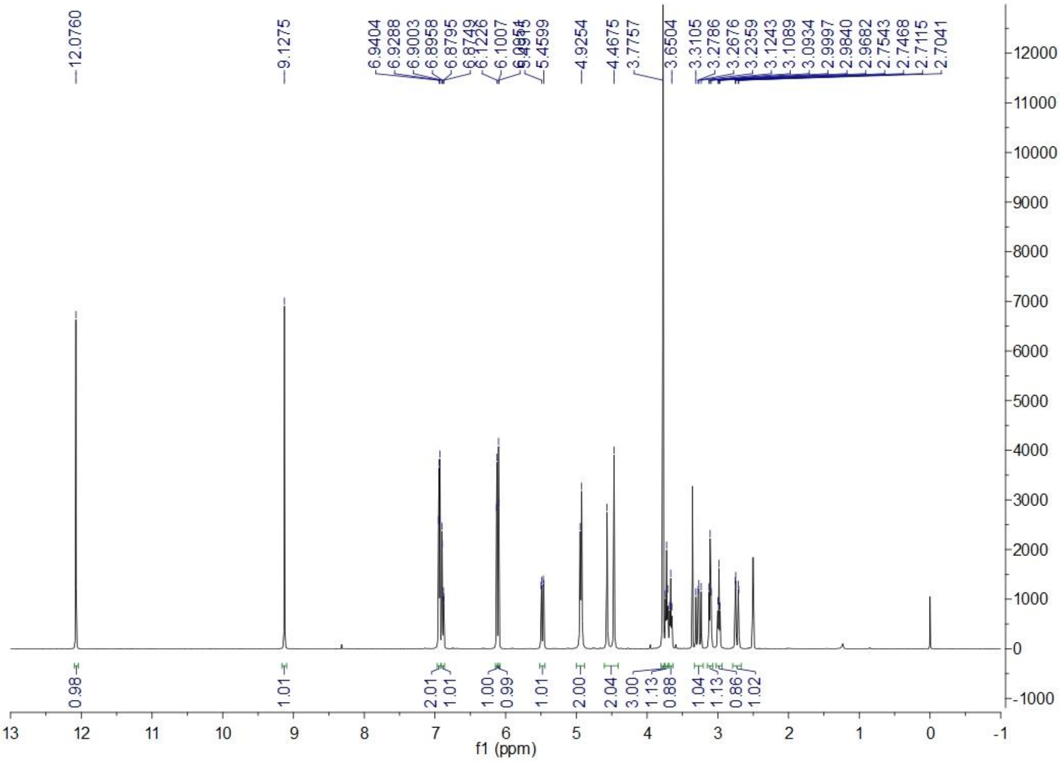

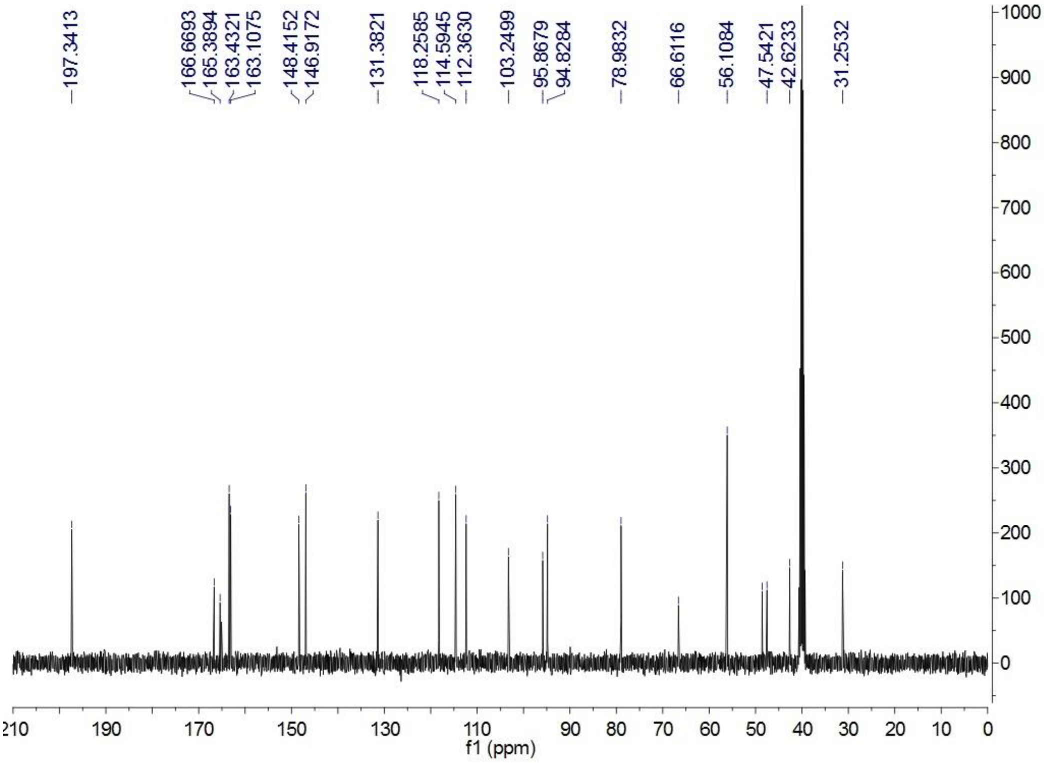

Figure S18. HRMS, ^{1}H NMR and ^{13}C NMR spectra for the target compound **6a:**

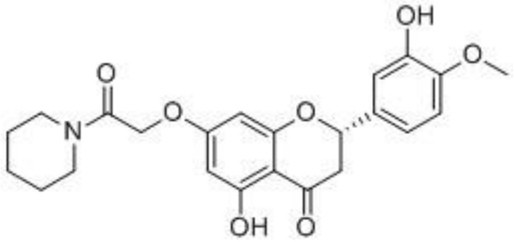

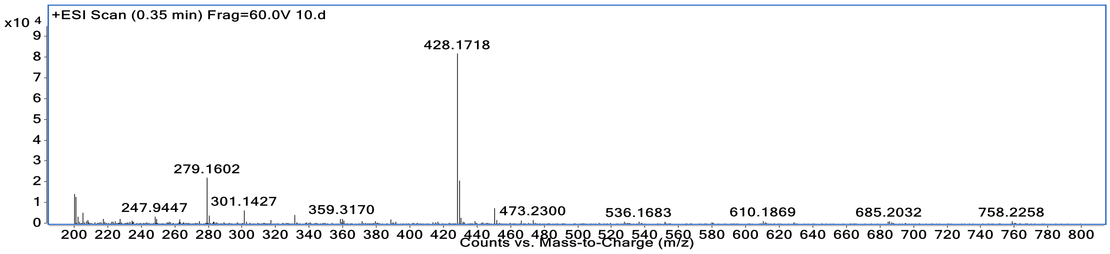

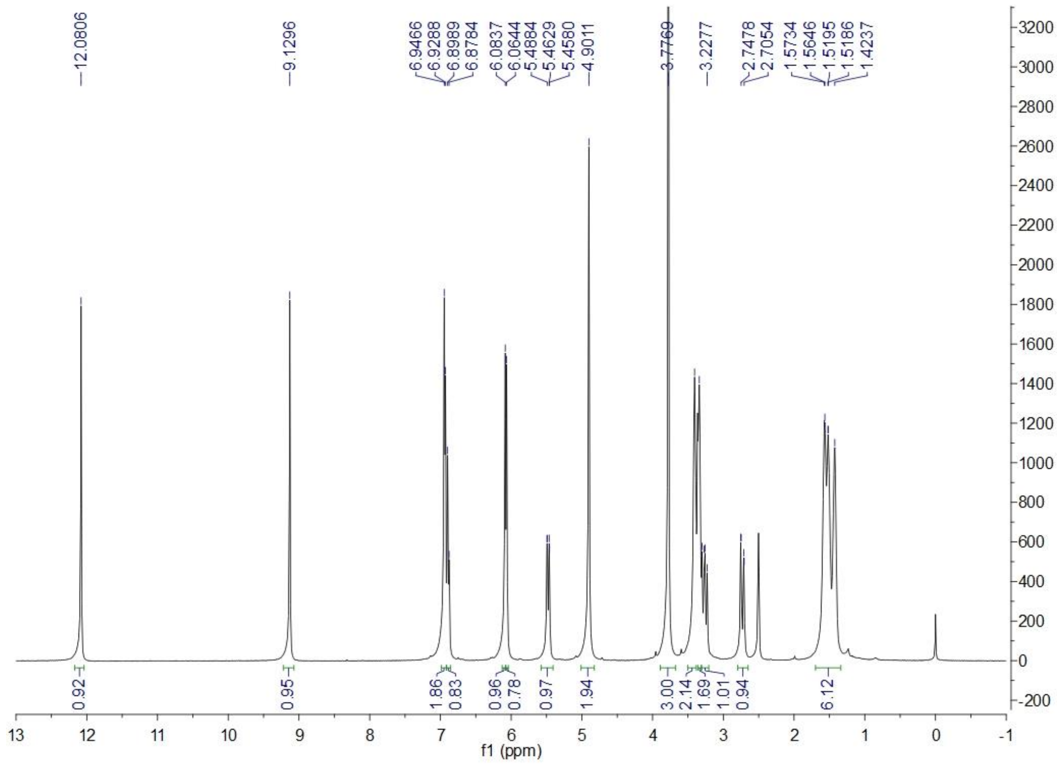
12.0806
9.1296
6.9466
6.9288
6.8989
6.8784
6.0837
6.0644
5.4884
5.4629
5.4580
4.9011
3.7769
3.2277
2.7478
2.7054
1.5734
1.5646
1.5195
1.5186
1.4237
0.92
0.95
1.86
0.83
0.96
0.78
0.97
1.94
3.00
2.14
1.69
1.01
0.94
6.12
f1 (ppm)

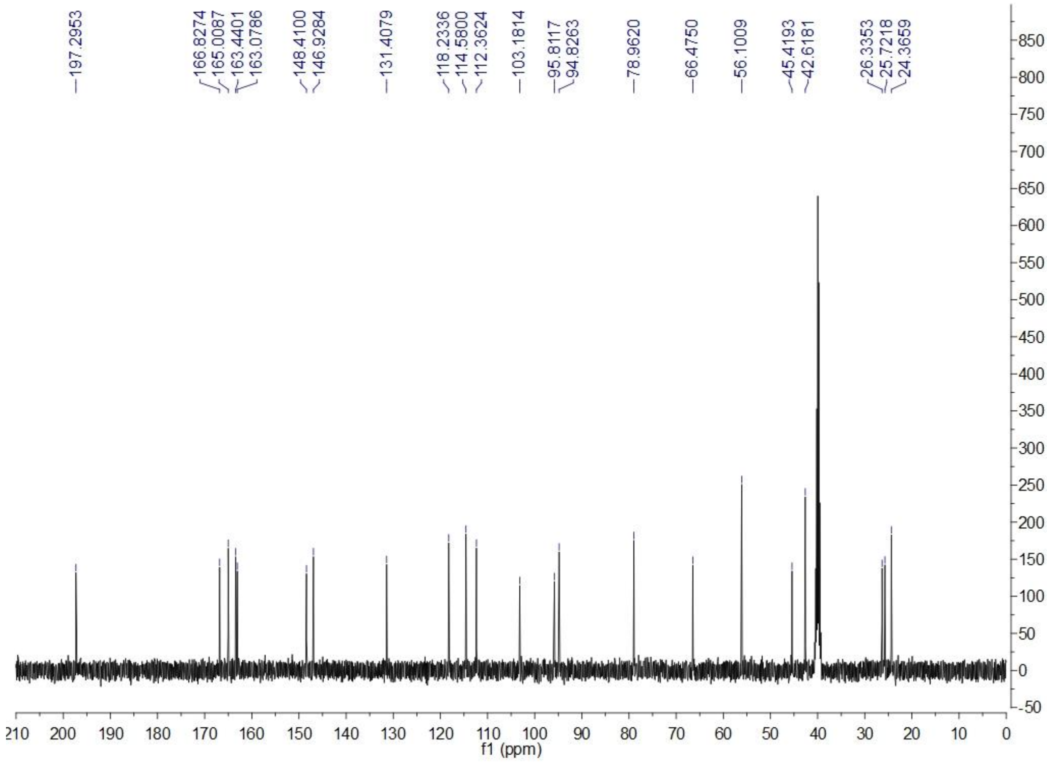
197.2953
166.8274
165.0087
163.4401
163.0786
148.4100
146.9284
131.4079
118.2336
114.5800
112.3624
103.1814
95.8117
94.8263
78.9620
66.4750
56.1009
45.4193
42.6181
26.3353
25.7218
24.3659
f1 (ppm)

Figure S19. HRMS, ^{1}H NMR and ^{13}C NMR spectra for the target compound **6b:**

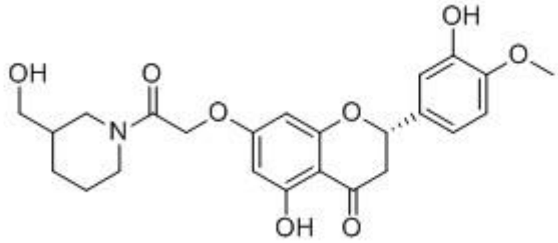

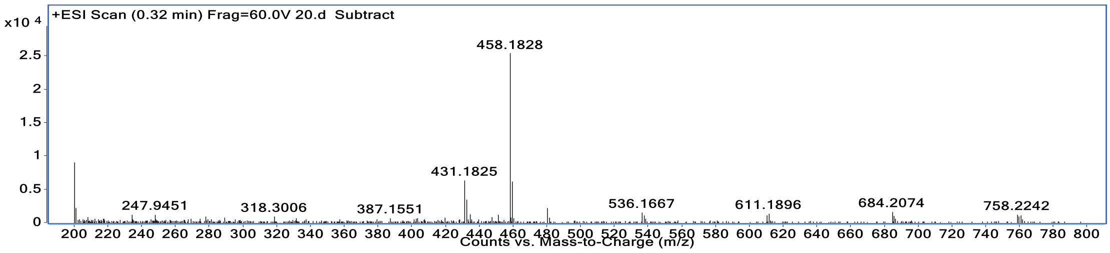

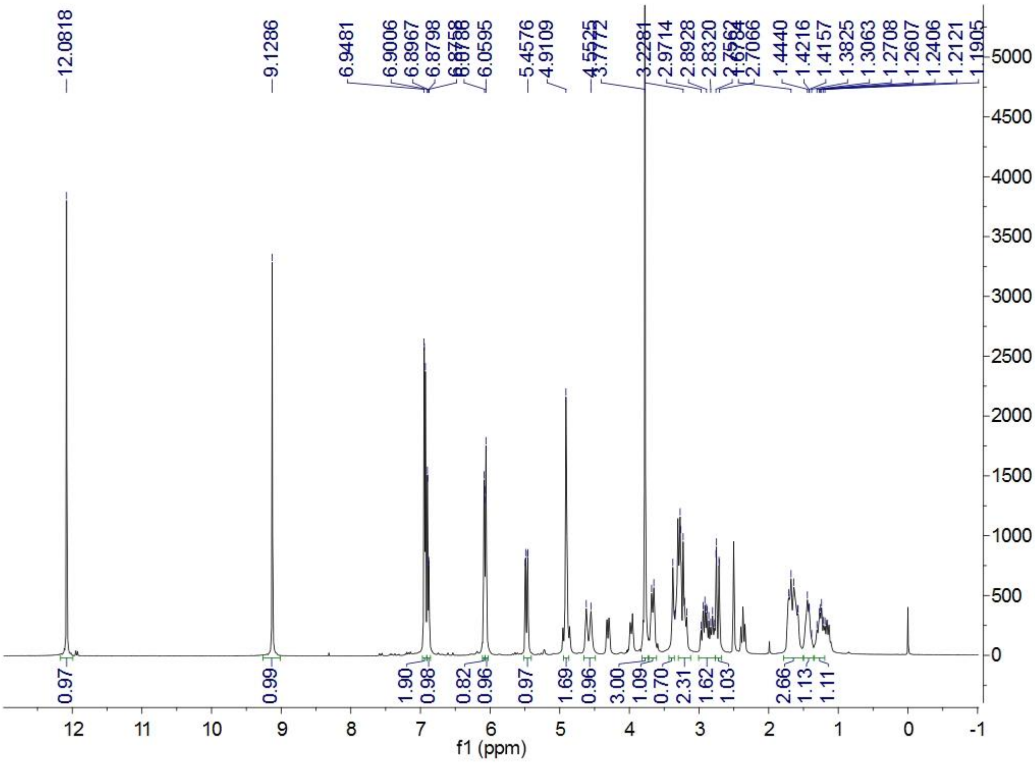

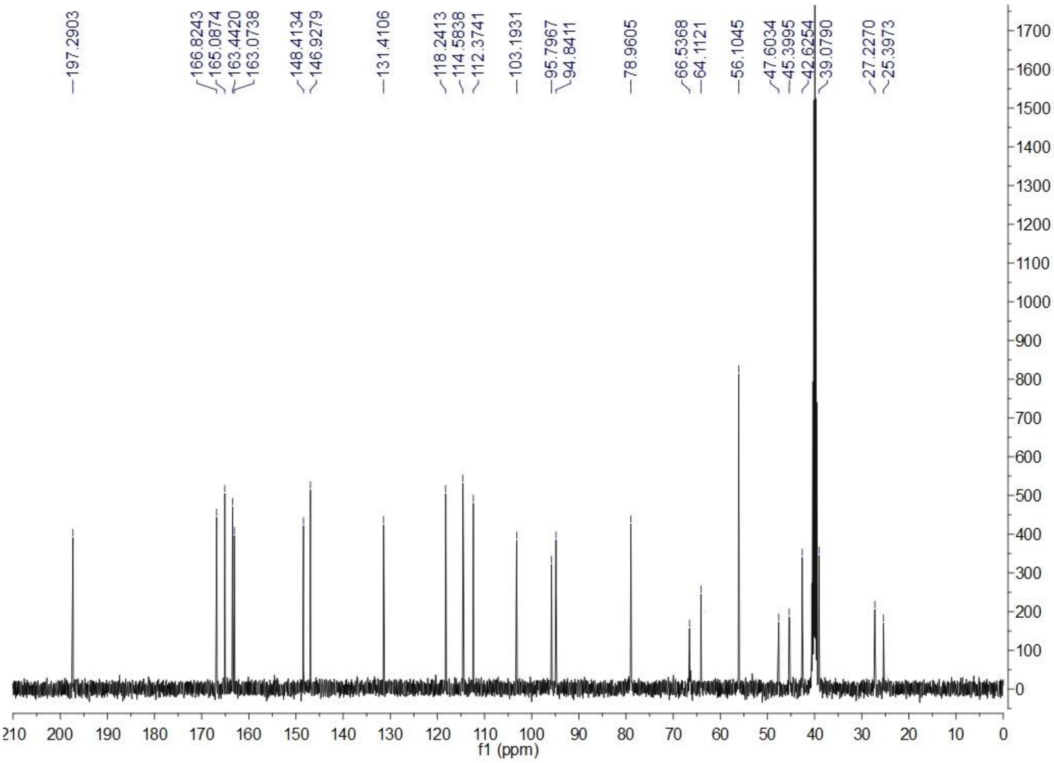

Figure S20. HRMS, ^{1}H NMR and ^{13}C NMR spectra for the target compound **6c:**

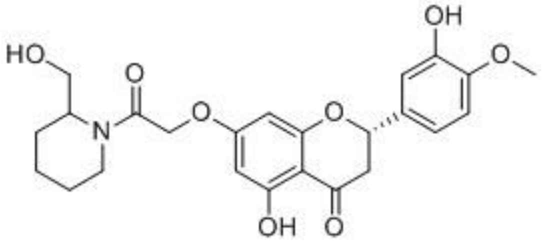

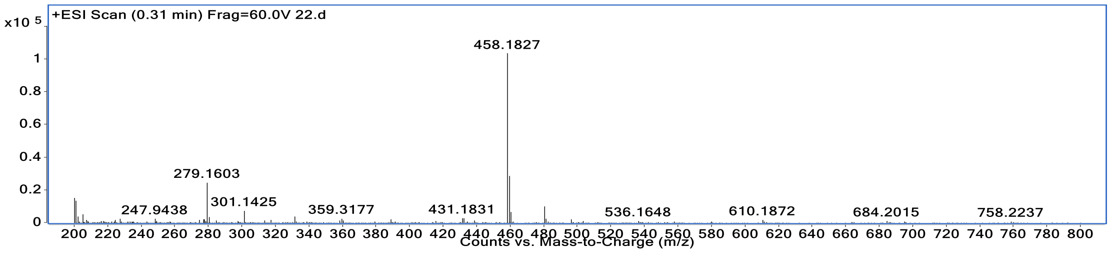

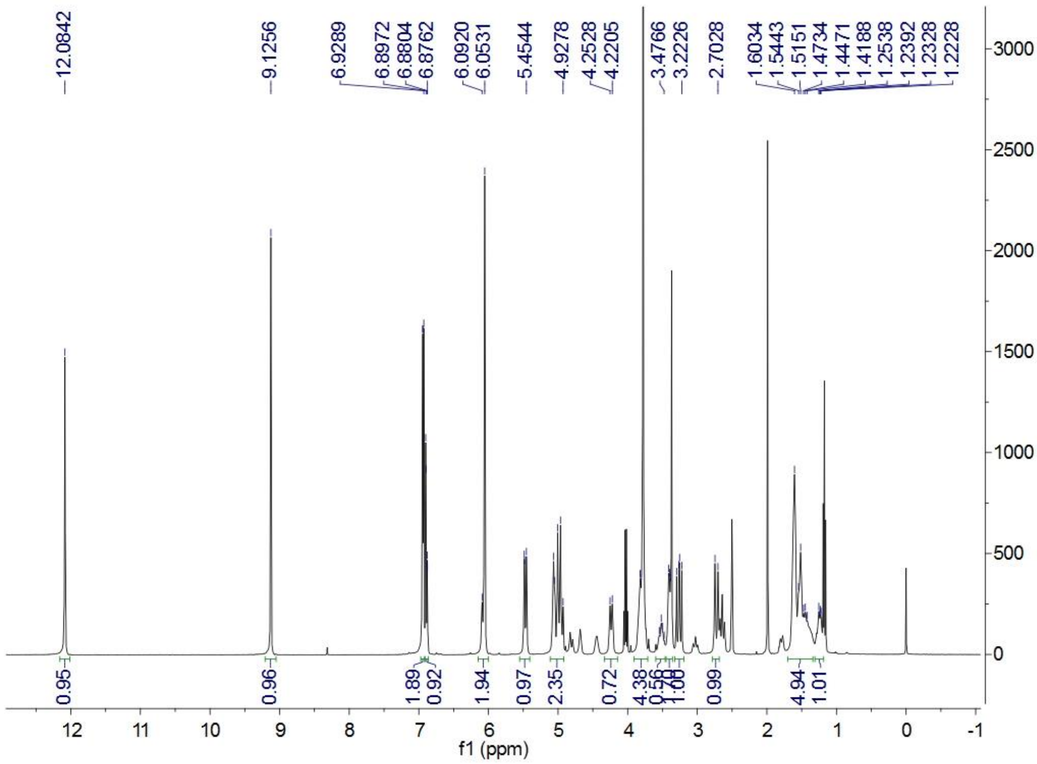
12.0842
9.1256
6.9289
6.8972
6.8804
6.8762
6.0920
6.0531
5.4544
4.9278
4.2528
4.2205
3.4766
3.2226
2.7028
1.6034
1.5443
1.5151
1.4734
1.4471
1.4188
1.2538
1.2392
1.2328
1.2228
0.95
0.96
1.89
0.92
1.94
0.97
2.35
0.72
4.38
2.70
0.99
4.94
1.01
f1 (ppm)

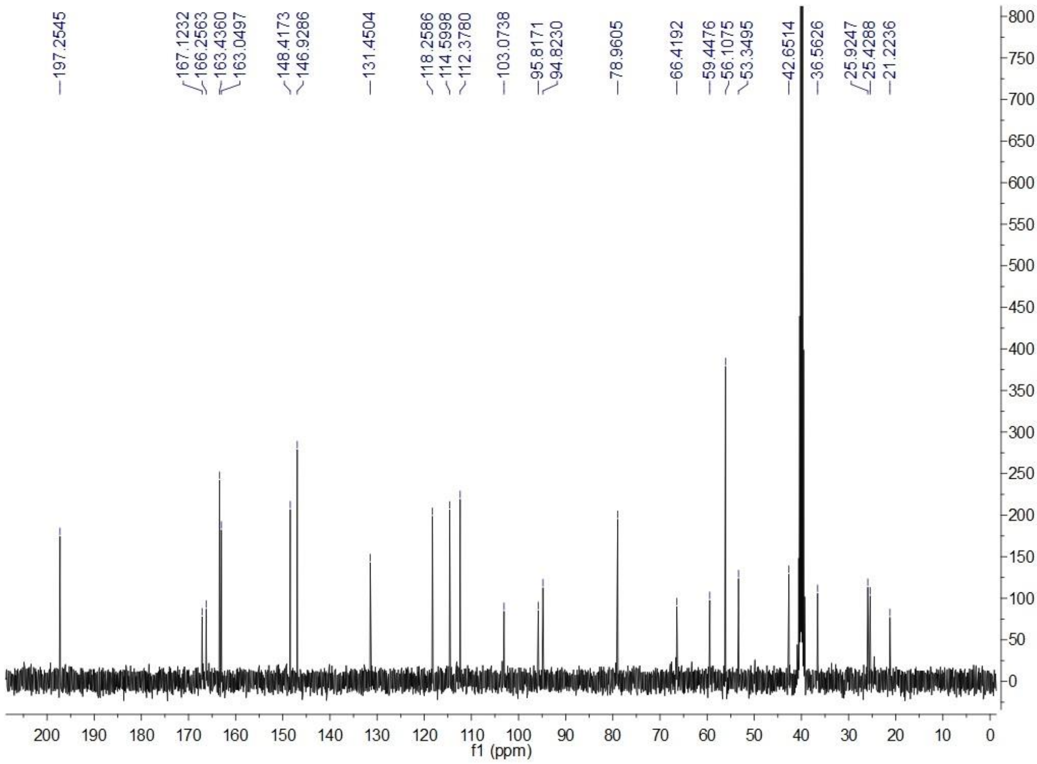
197.2545
167.1232
166.2563
163.4360
163.0497
148.4173
146.9286
131.4504
118.2586
114.5998
112.3780
103.0738
95.8171
94.8230
78.9605
66.4192
59.4476
56.1075
53.3495
42.6514
36.5626
25.9247
25.4288
21.2236
f1 (ppm)

Figure S21. HRMS, ^{1}H NMR and ^{13}C NMR spectra for the target compound **6d:**

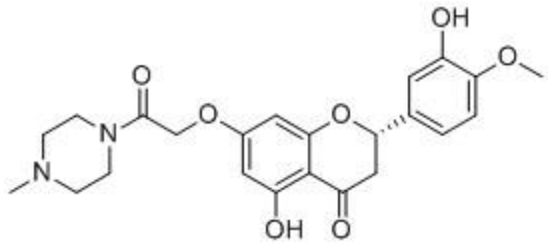

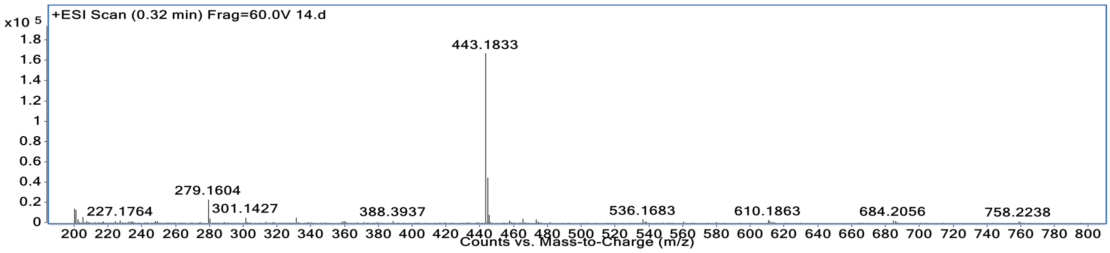

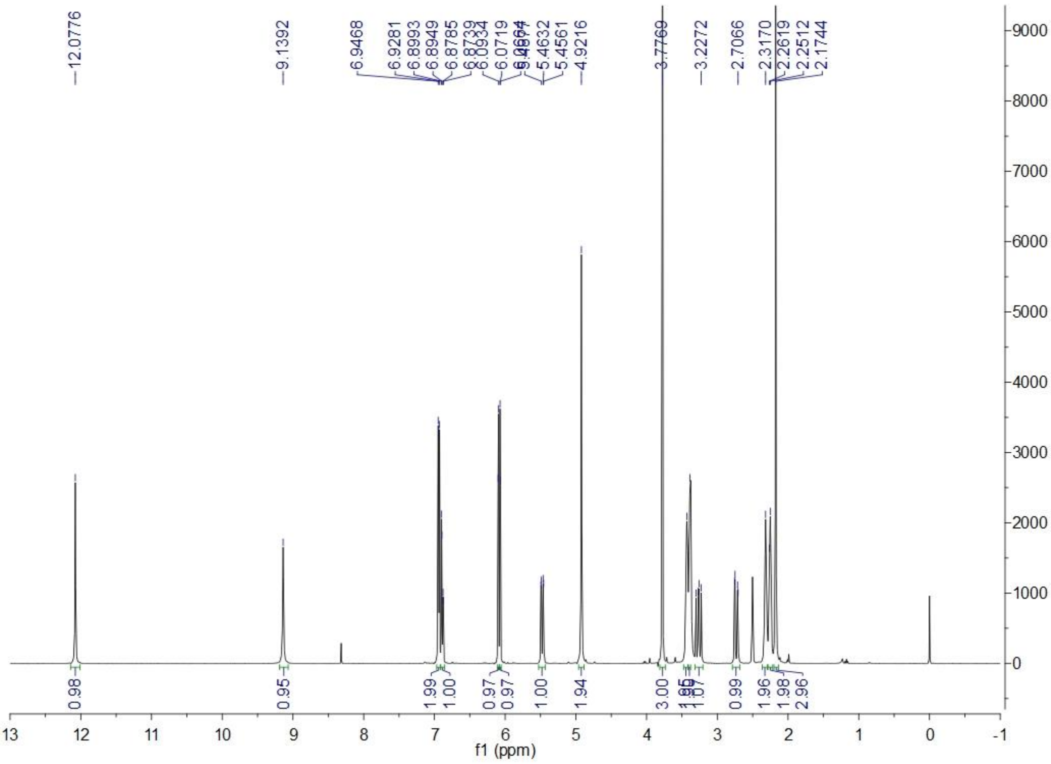

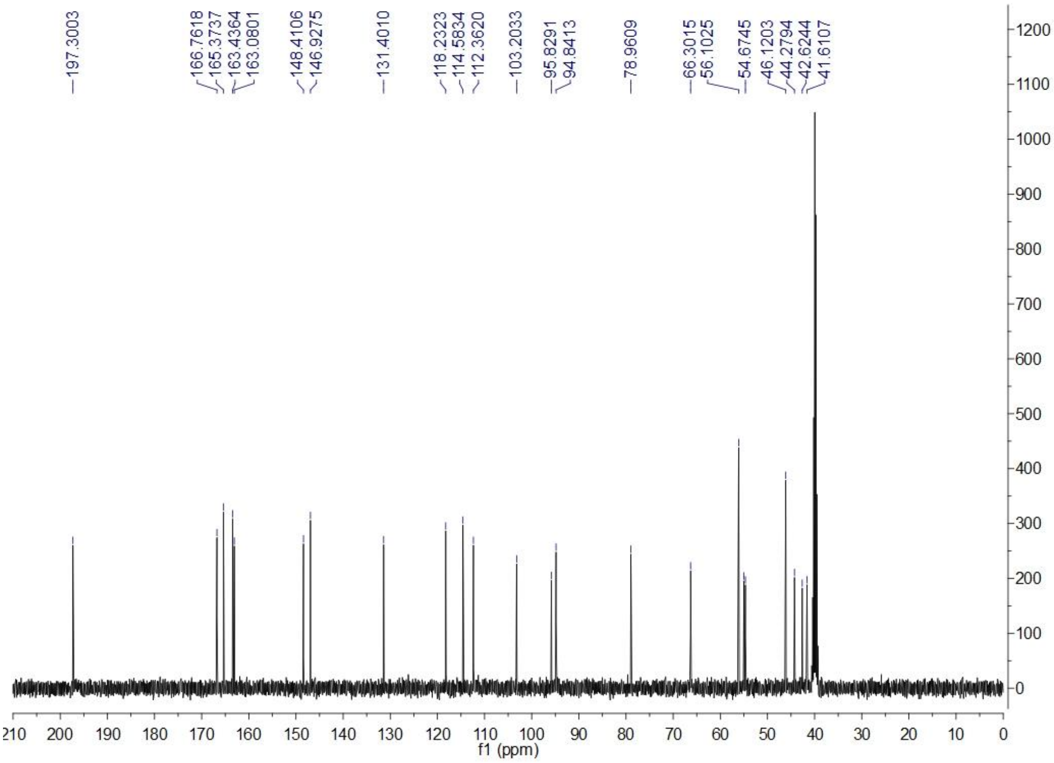

Figure S22. HRMS, ^{1}H NMR and ^{13}C NMR spectra for the target compound **6e:**

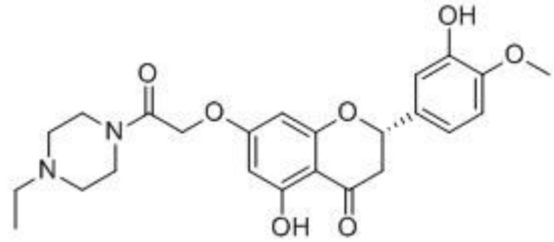

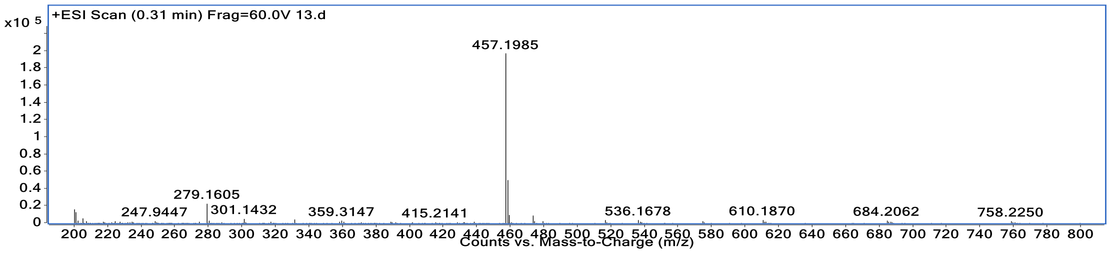

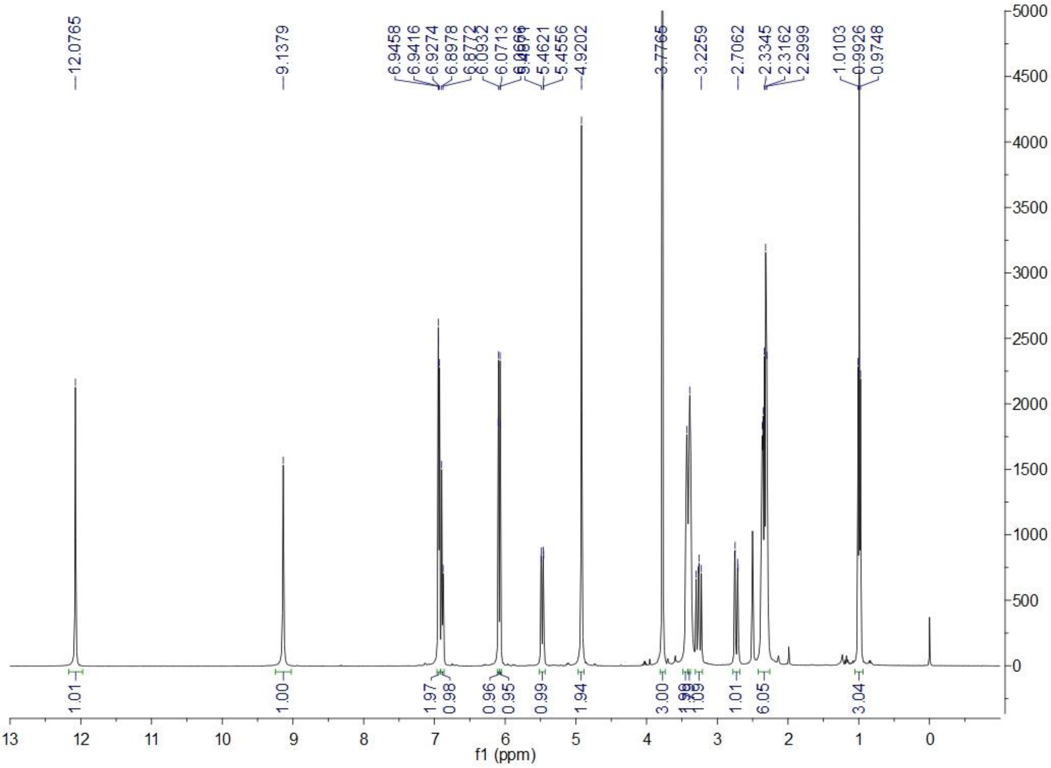

12.0765
9.1379
6.9458
6.9416
6.9274
6.8978
6.8772
6.0932
6.0713
5.4621
5.4556
4.9202
3.7765
3.2259
2.7062
2.3345
2.3162
2.2999
1.0103
0.9926
0.9748
1.01
1.00
1.97
0.98
0.96
0.95
0.99
1.94
3.00
1.09
1.01
6.05
3.04
f1 (ppm)

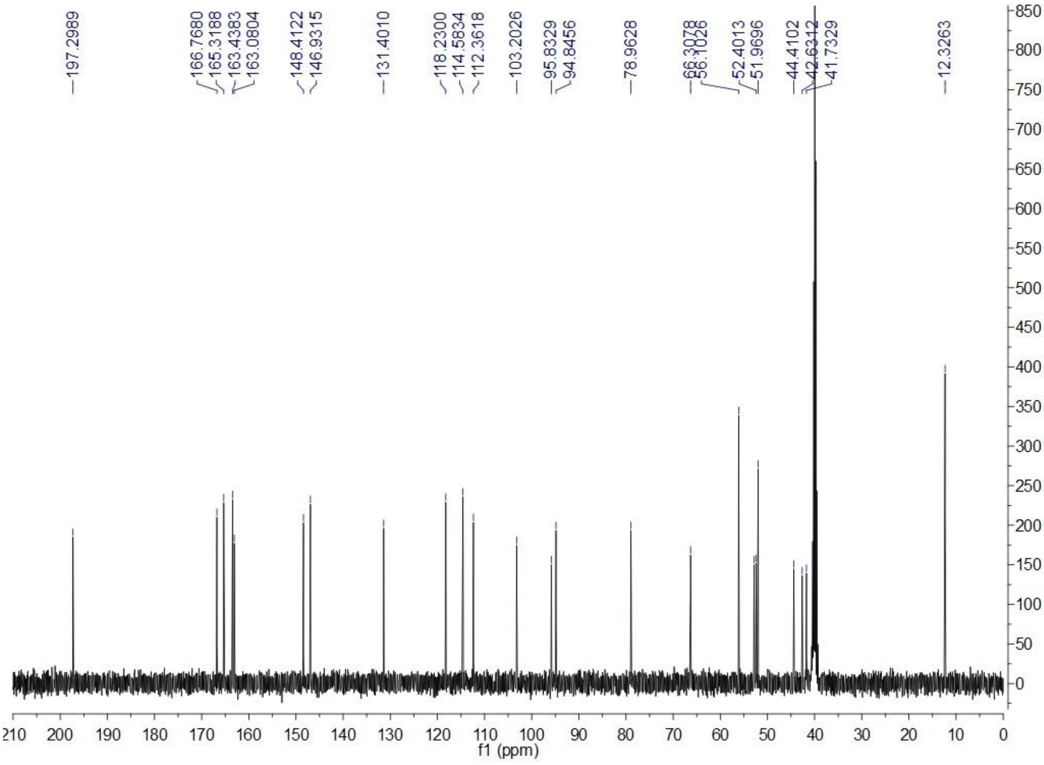

197.2989
166.7680
165.3188
163.4383
163.0804
148.4122
146.9315
131.4010
118.2300
114.5834
112.3618
103.2026
95.8329
94.8456
78.9628
66.3078
56.1026
52.4013
51.9696
44.4102
42.6312
41.7329
12.3263
f1 (ppm)

Figure S23. HRMS, ^{1}H NMR and ^{13}C NMR spectra for the target compound **6f:**

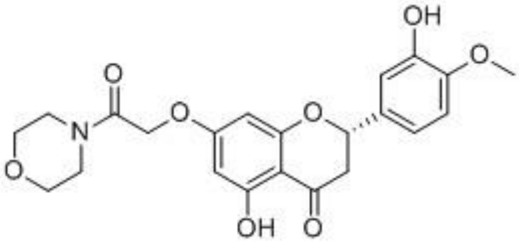

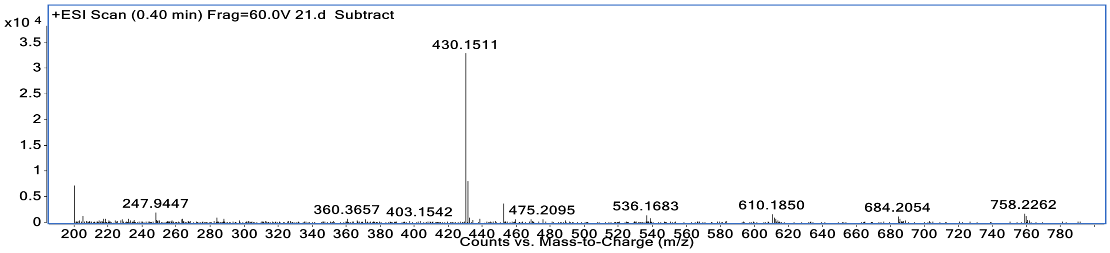

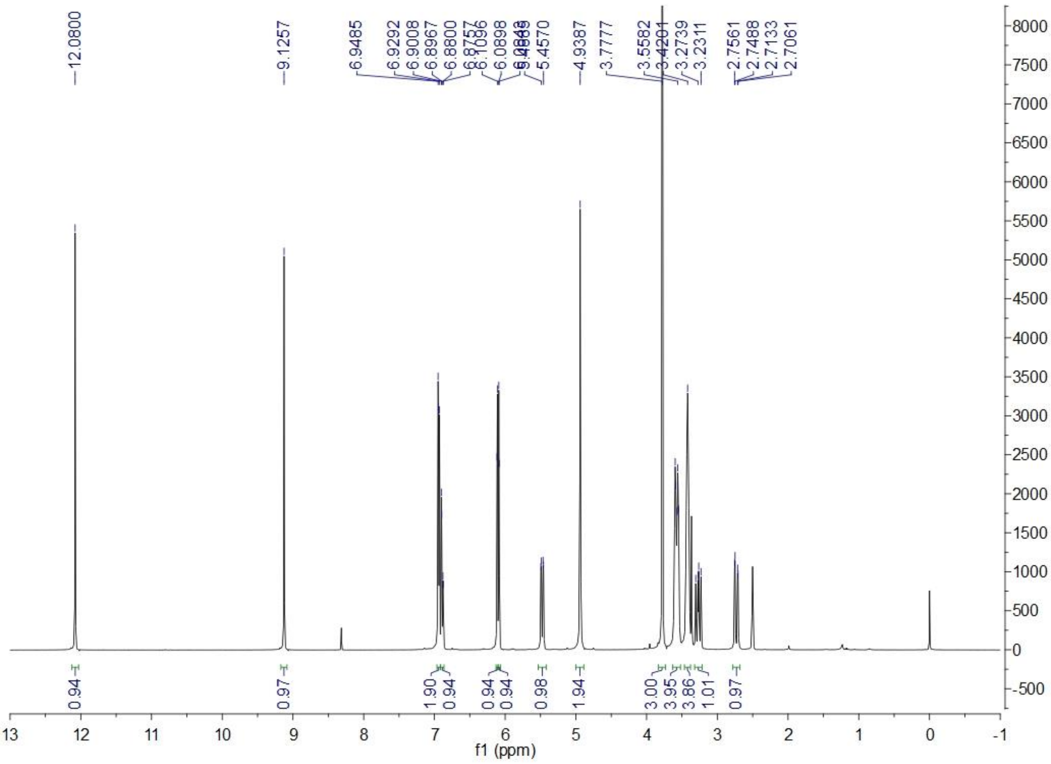

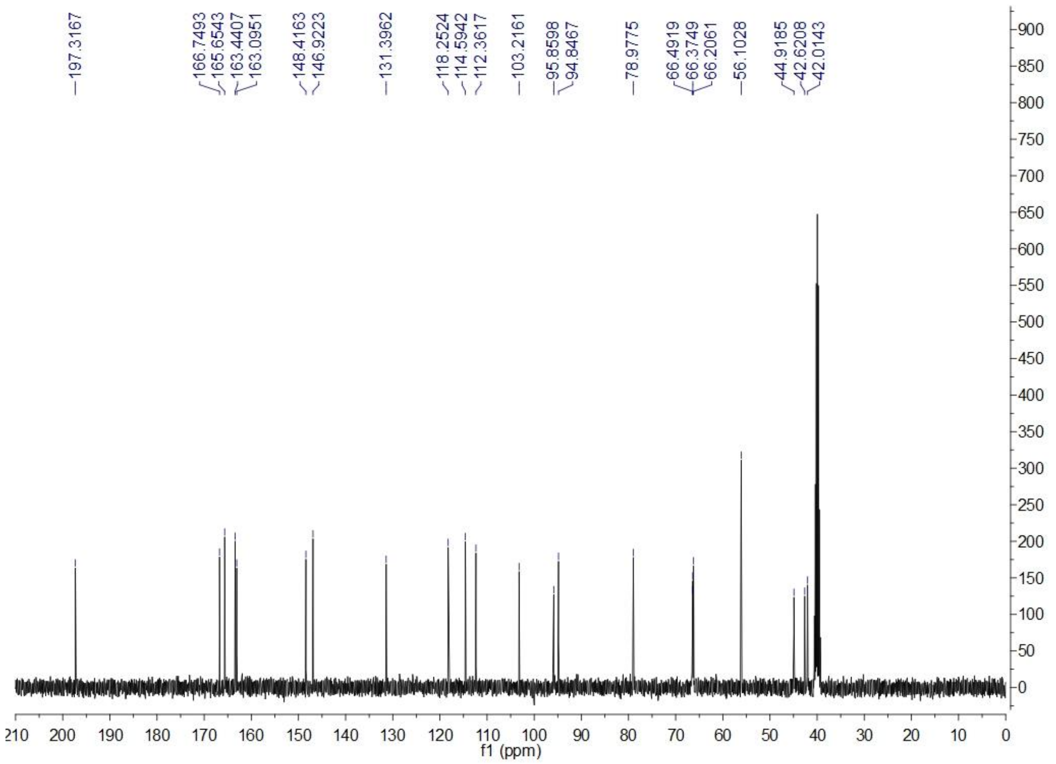

Figure S24. HRMS, ^{1}H NMR and ^{13}C NMR spectra for the target compound **7a:**

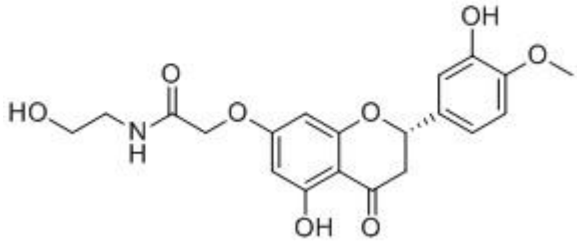

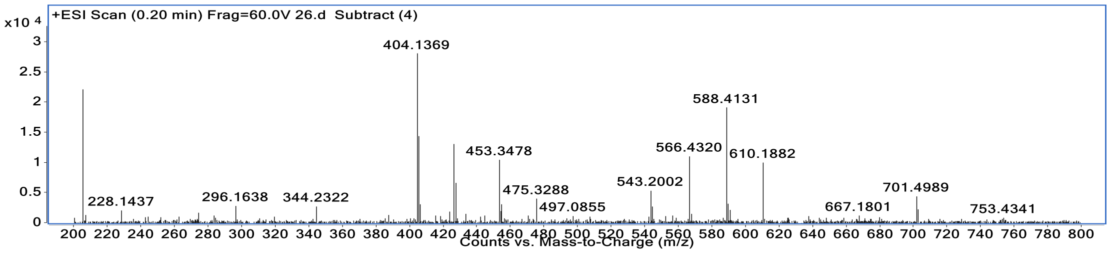

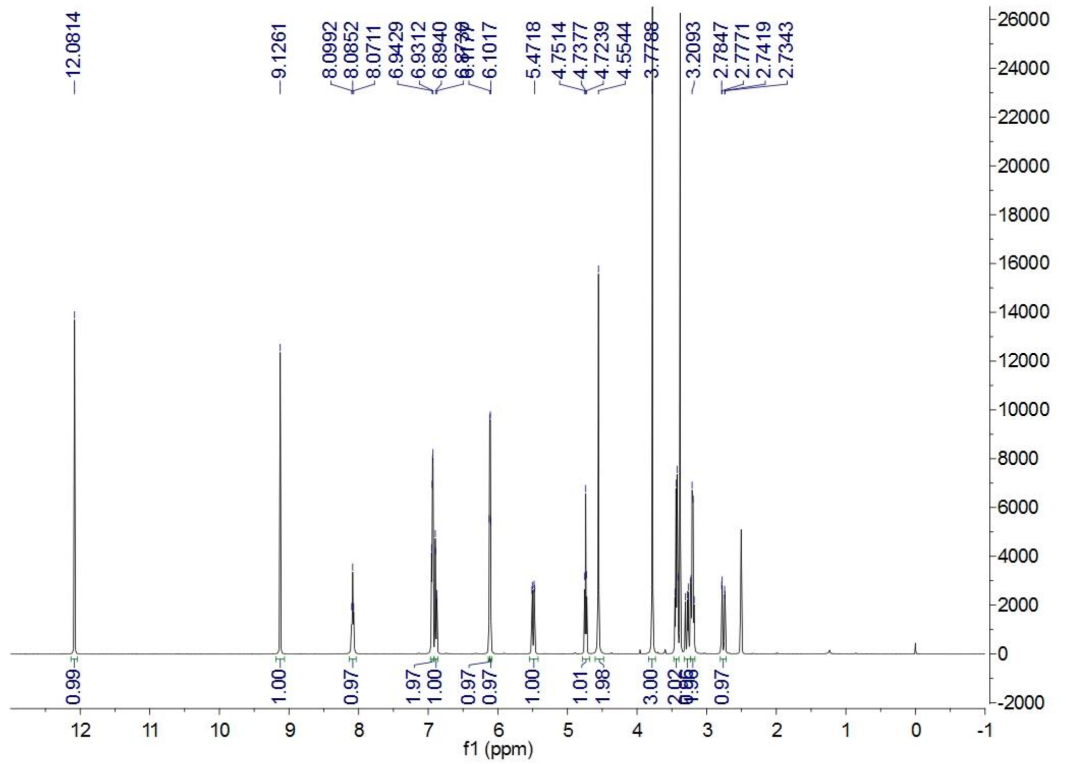
12.0814
9.1261
8.0992
8.0852
8.0711
6.9429
6.9312
6.8940
6.1017
5.4718
4.7514
4.7377
4.7239
4.5544
3.7788
3.2093
2.7847
2.7771
2.7419
2.7343
0.99
1.00
0.97
1.97
1.00
0.97
0.97
1.00
1.01
1.98
3.00
2.02
1.98
0.97
f1 (ppm)

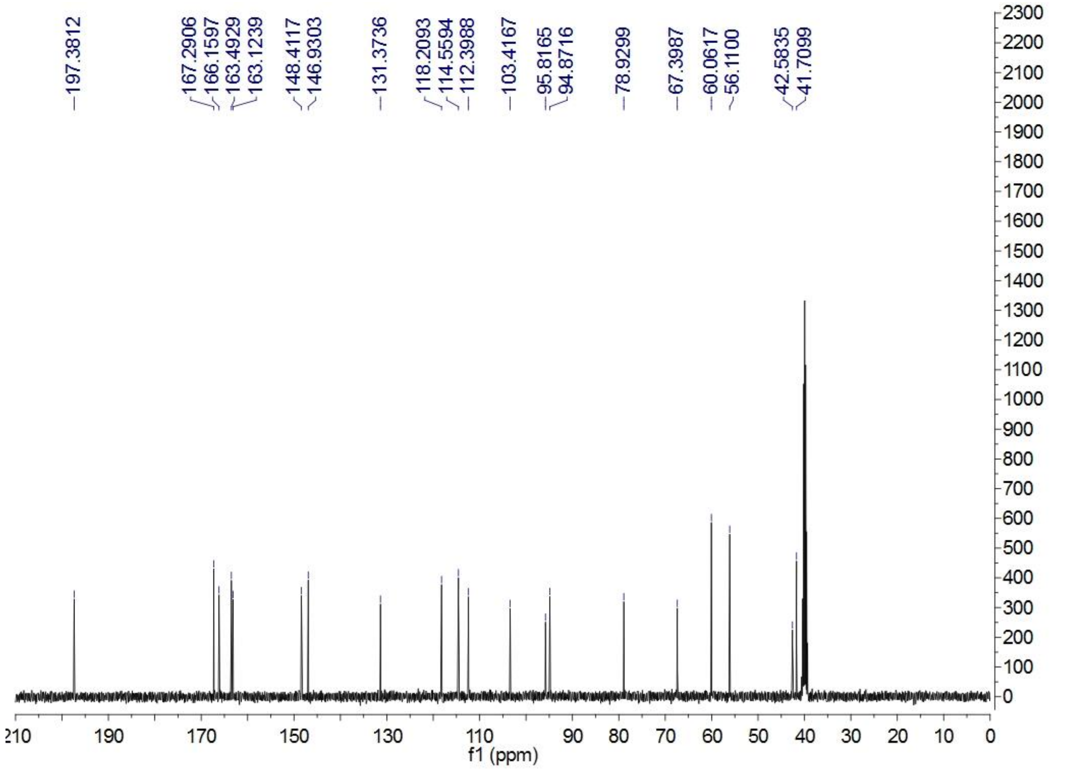
197.3812
167.2906
166.1597
163.4929
163.1239
148.4117
146.9303
131.3736
118.2093
114.5594
112.3988
103.4167
95.8165
94.8716
78.9299
67.3987
60.0617
56.1100
42.5835
41.7099
f1 (ppm)

Figure S25. HRMS, ^{1}H NMR and ^{13}C NMR spectra for the target compound **7b:**

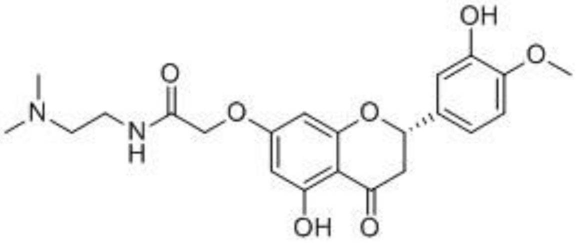

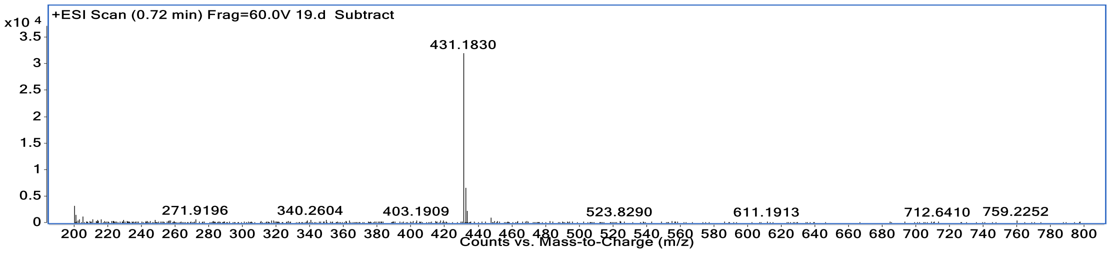

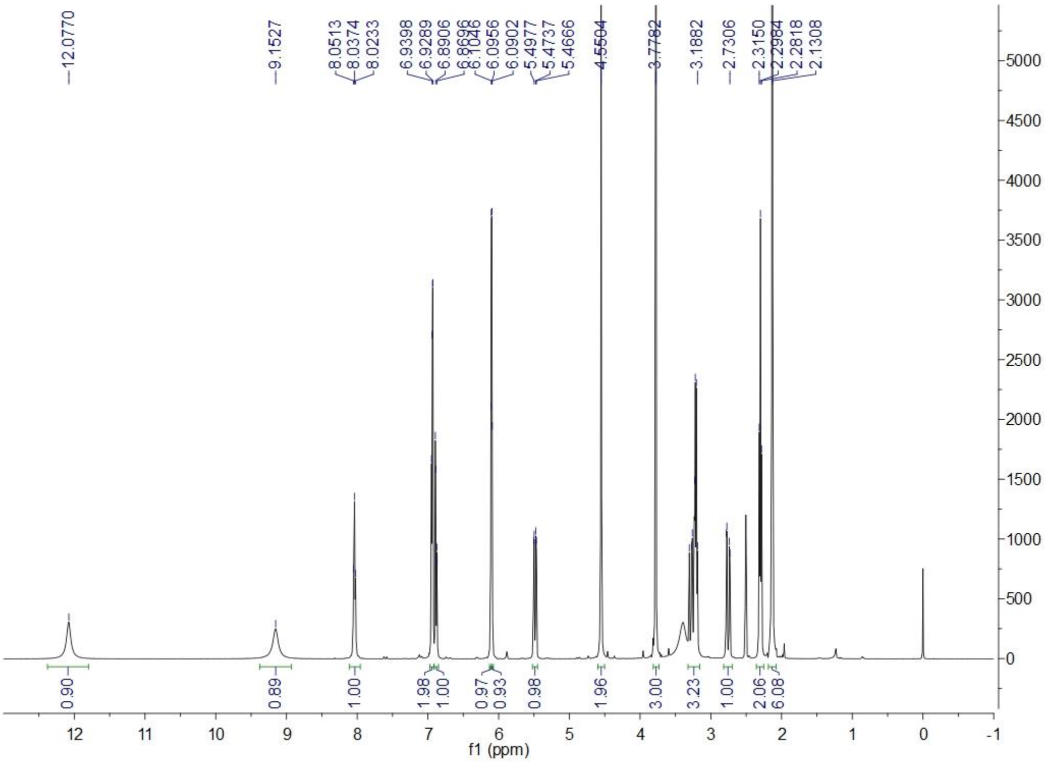

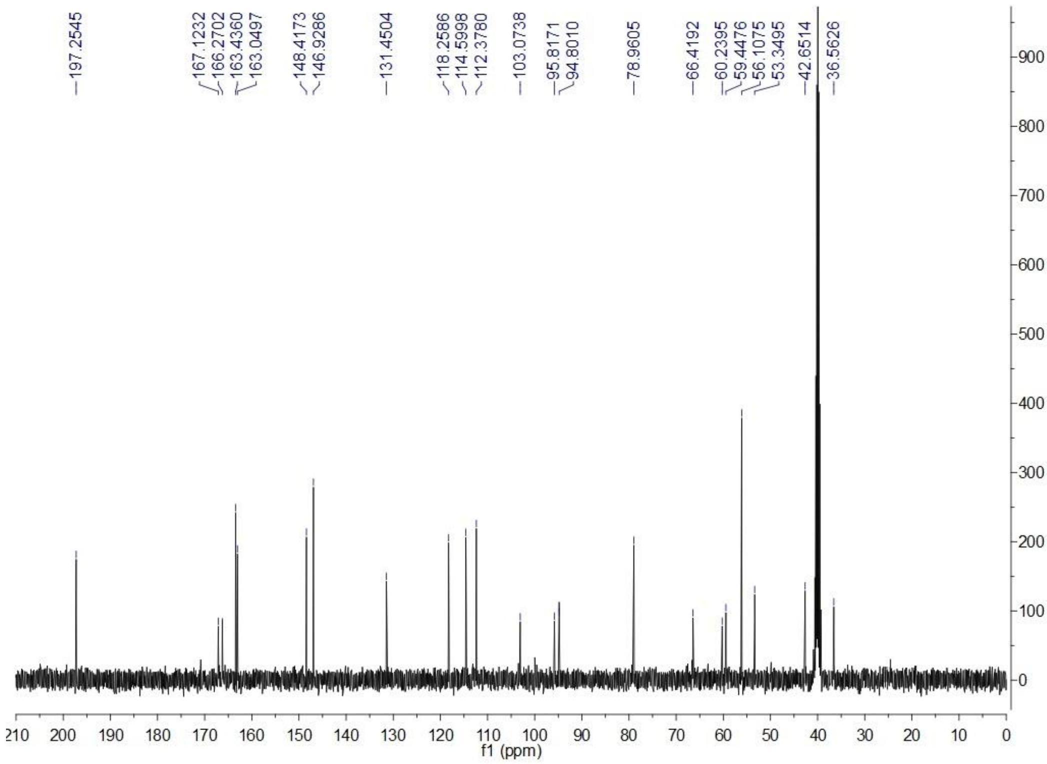

Figure S26. HRMS, ^{1}H NMR and ^{13}C NMR spectra for the target compound **7c:**

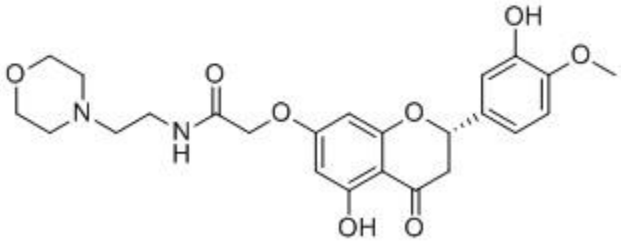

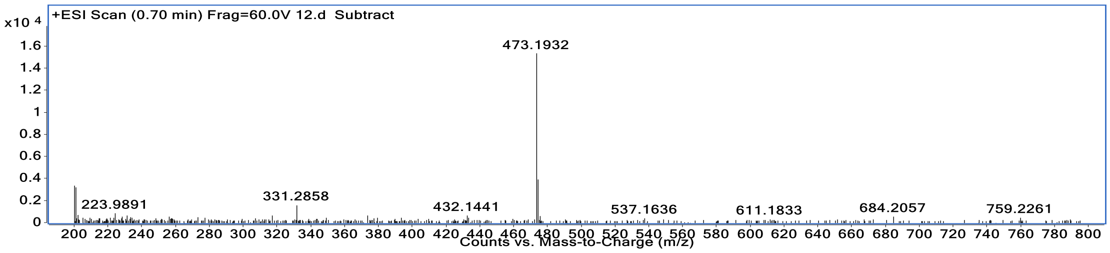

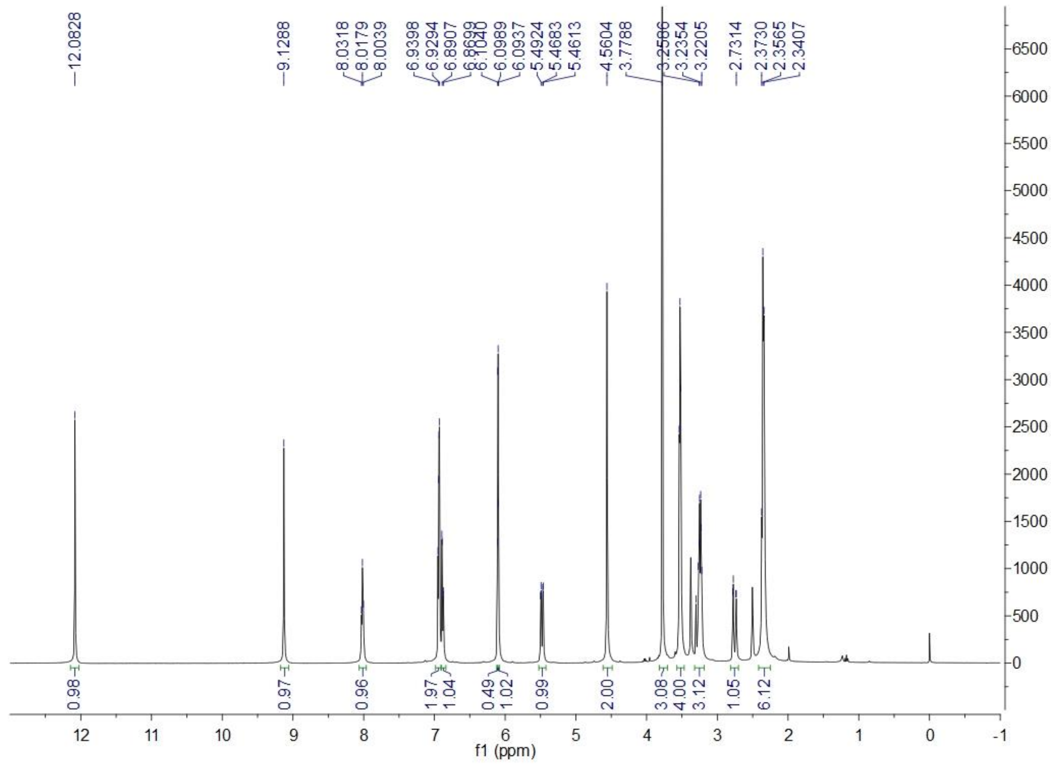
12.0828
9.1288
8.0318
8.0179
8.0039
6.9398
6.9294
6.8907
6.0989
6.0937
5.4924
5.4683
5.4613
4.5604
3.7788
3.2354
3.2205
2.7314
2.3730
2.3565
2.3407
0.98
0.97
0.96
1.97
1.04
0.49
1.02
0.99
2.00
3.08
4.00
3.12
1.05
6.12
f1 (ppm)

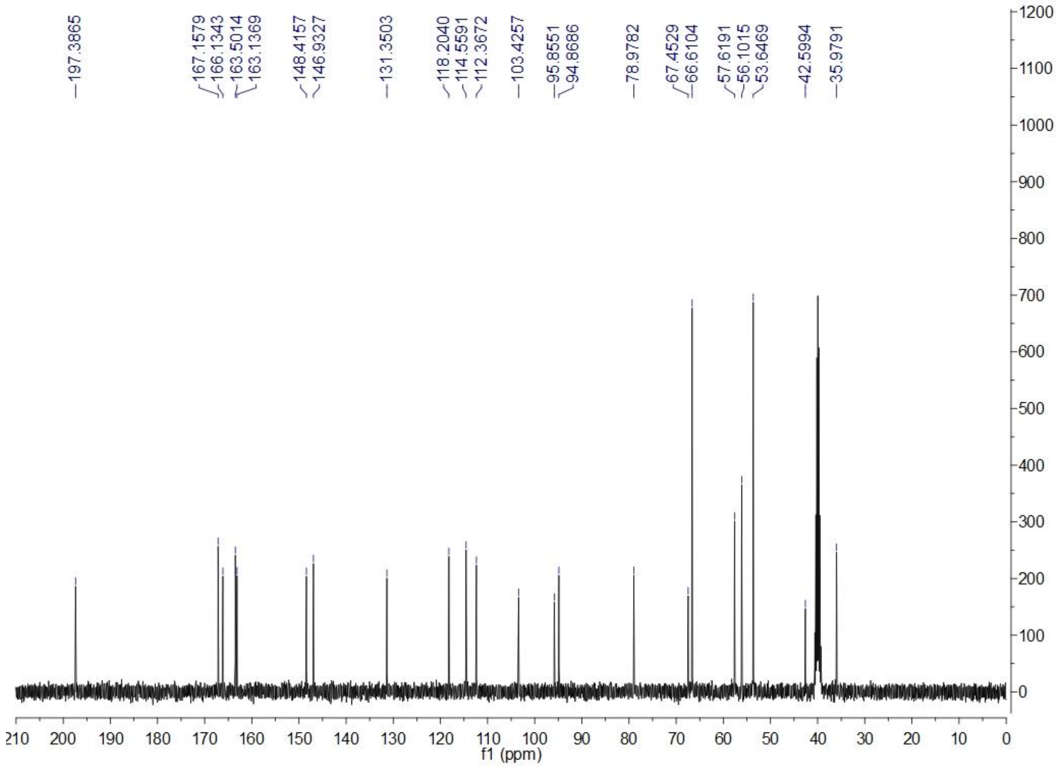
197.3865
167.1579
166.1343
163.5014
163.1369
148.4157
146.9327
131.3503
118.2040
114.5591
112.3672
103.4257
95.8551
94.8686
78.9782
67.4529
66.6104
57.6191
56.1015
53.6469
42.5994
35.9791
f1 (ppm)
